# Involvement of HIF-1α in the Detection, Signaling, and Repair of DNA Double-Strand Breaks after Photon and Carbon-Ion Irradiation

**DOI:** 10.3390/cancers13153833

**Published:** 2021-07-30

**Authors:** Anne-Sophie Wozny, Arnaud Gauthier, Gersende Alphonse, Céline Malésys, Virginie Varoclier, Michael Beuve, Delphine Brichart-Vernos, Nicolas Magné, Nicolas Vial, Dominique Ardail, Tetsuo Nakajima, Claire Rodriguez-Lafrasse

**Affiliations:** 1 Cellular and Molecular Radiobiology Laboratory, Lyon-Sud Medical School, UMR CNRS5822/IP2I, Univ Lyon, Lyon 1 University, 69921 Oullins, France; Anne-Sophie.Wozny@univ-lyon1.fr (A.-S.W.); Arnaud.Gauthier@univ-lyon1.fr (A.G.); Gersende.Alphonse@univ-lyon1.fr (G.A.); Celine.Malesys@univ-lyon1.fr (C.M.); Virginie.Varoclier@univ-lyon1.fr (V.V.); Delphine.Vernos@univ-lyon1.fr (D.B.-V.); Nicolas.Magne@icloire.fr (N.M.); Nicolas.Vial@icloire.fr (N.V.); Dominique.Ardail@univ-lyon1.fr (D.A.); 2 Department of Biochemistry and Molecular Biology, Lyon-Sud Hospital, Hospices Civils de Lyon, 69310 Pierre-Bénite, France; 3 Univ Lyon, Lyon 1 University, UMR CNRS5822/IP2I, 69100 Villeurbanne, France; Michael.Beuve@univ-lyon1.fr; 4 Department of Radiotherapy, Institute of Cancerology Lucien Neuwirth, 42270 Saint-Priest-en-Jarez, France; 5 Department of Radiation Effects Research, National Institute of Radiological Sciences, National Institute for Quantum and Radiological Science and Technology, Chiba 263-8555, Japan; nakajima.tetsuo@qst.go.jp

**Keywords:** hypoxia-inducible factor 1, double-strand breaks, DNA repair, homologous recombination, non-homologous end joining pathway, irradiations, carbon ions, photons, hypoxia, cancer stem cells

## Abstract

**Simple Summary:**

Hypoxia-Inducible Factor 1α (HIF-1α), the main regulator of the oxygen homeostasis, promotes cancer cell survival through proliferation, angiogenesis, metastasis and radioresistance. Previously, our group demonstrated that silencing HIF-1α under hypoxia leads to a substantial radiosensitization of Head-and-Neck Squamous Cell Carcinoma (HNSCC) cells after both photons and carbon-ions, probably resulting from an accumulation of deleterious complex DNA damage. In this study, we aimed at determining the potential role of HIF-1α in the detection, signaling, and repair of DNA Double-Strand-Breaks (DSBs) in response to both irradiations, under hypoxia, in two HNSCC cell lines and their subpopulations of Cancer-Stem Cells (CSCs). Silencing HIF-1α under hypoxia led us to demonstrate the involvement of this transcriptional regulator in DSB repair in non-CSCS and CSC, thus highlighting its targeting together with radiation as a promising therapeutic strategy against radioresistant tumor cells in hypoxic niches.

**Abstract:**

Hypoxia-Inducible Factor 1α (HIF-1α), which promotes cancer cell survival, is the main regulator of oxygen homeostasis. Hypoxia combined with photon and carbon ion irradiation (C-ions) stabilizes HIF-1α. Silencing HIF-1α under hypoxia leads to substantial radiosensitization of Head-and-Neck Squamous Cell Carcinoma (HNSCC) cells after both photons and C-ions. Thus, this study aimed to clarify a potential involvement of HIF-1α in the detection, signaling, and repair of DNA Double-Strand-Breaks (DSBs) in response to both irradiations, in two HNSCC cell lines and their subpopulations of Cancer-Stem Cells (CSCs). After confirming the nucleoshuttling of HIF-1α in response to both exposure under hypoxia, we showed that silencing HIF-1α in non-CSCs and CSCs decreased the initiation of the DSB detection (P-ATM), and increased the residual phosphorylated H2AX (γH2AX) foci. While HIF-1α silencing did not modulate 53BP1 expression, P-DNA-PKcs (NHEJ-c) and RAD51 (HR) signals decreased. Altogether, our experiments demonstrate the involvement of HIF-1α in the detection and signaling of DSBs, but also in the main repair pathways (NHEJ-c and HR), without favoring one of them. Combining HIF-1α silencing with both types of radiation could therefore present a potential therapeutic benefit of targeting CSCs mostly present in tumor hypoxic niches.

## 1. Introduction

In solid tumors, hypoxia has been extensively described as a factor of radioresistance associated with higher survival rate, induction of metastasis, genetic instability, and poor prognosis [1,2,3]. Furthermore, hypoxia favors anaerobic glycolysis over oxidative phosphorylation, leading to acidification of the inner parts of the tumor [4]. Under hypoxic conditions, this metabolic switch contributes to apoptosis resistance and facilitates tumor cell proliferation. Several oxygen sensors have been described, such as the changes in the chromatin conformation that affect the transcriptional activity, the release of the reactive oxygen species (ROS) by the mitochondria, the inhibition of the mammalian target of the rapamycin (mTOR) signaling, or the activation of the reticulum endoplasmic stress sensors through the unfolded protein response (UPR) pathway. However, the best-understood sensing mechanism for oxygen concentrations relies on the HIF family of transcription factors [4]. Hypoxia-Inducible Factor 1 (HIF-1) is the most prominent transcription factor of the cellular response to hypoxia. It plays a critical role in mammalian development, but also in promoting cancer progression through survival, angiogenesis, or epithelial-to-mesenchymal transition [5,6,7,8]. HIF-1 is a heterodimeric protein composed of the constitutive subunit HIF-1β and the oxygen-regulated subunit HIF-1α [9,10,11]. HIF-1α is tightly regulated by cellular oxygen concentration and is hydroxylated on two proline residues under normoxia by propyl-hydroxylase [6]. This enables its binding to Von Hippel-Lindau protein and the recruitment of E3 ubiquitin-protein ligase, leading to its poly-ubiquitination and degradation by the proteasome [8,12,13]. Under hypoxia, HIF-1α translocates to the nucleus and is dimerized with HIF-1β [13,14]. The complex binds to hypoxia-responsive elements and activates the transcription of genes, which facilitate the metabolic adaptation to hypoxia but also cancer progression through immune evasion or stem-cell maintenance [13,14]. Due to the presence of hypoxic tumoral niches, HIF-1α is overexpressed in the majority of solid cancers, making it an attractive therapeutic target [8,15]. Although many factors contribute to HIF-1α stabilization, ROS, produced homogeneously by photon irradiation, have been more recently involved in this mechanism, with the fastest stabilization of HIF-1α in Cancer Stem Cells (CSCs) compared to non-CSCs [8,13,16]. Alternatively, carbon ion irradiations (C-ions), which present a better ballistic precision and a higher biological efficiency, less dependent on the oxygen concentration, do not induce stabilization of HIF-1α [16,17,18,19,20]. ROS are produced and concentrated only in the tracks of ions, and their levels are probably not sufficient to reach a threshold necessary to stabilize HIF1-α and activate its relative signaling pathways, a phenomenon described as the stealth bomber effect [20]. However, silencing HIF1-α under hypoxia induced a radiosensitization of HNSCC (Head-and-Neck Squamous Cell Carcinoma) cell lines and their CSC subpopulation either with photons or C-ions [16]. Indeed, C-ions have also been reported to better kill cancer cells, and particularly CSCs, by inducing clustered DNA Double-Strand Breaks (DSBs), which are extremely complex to repair [21,22,23]. Once induced, DSBs are first signalized by the phosphorylation of the ataxia-telangiectasia mutated protein (P-ATM) and the subsequent activation of the histone H2AX (γH2AX) [24,25]. Then, the two major DSB repair pathways, i.e., the canonical non-homologous end-joining (NHEJ-c) and the homologous-recombination (HR) pathways, are activated [26]. The error-prone NHEJ-c involves Ku70/80, which recognizes the DSB ends, induces the recruitment of DNA-PKcs and its phosphorylation by ATM. Subsequently, the XRCC4-DNA ligase IV complex ligates the two DNA ends. The phosphorylation of ATM and H2AX also activates the P53-binding protein 1 (53BP1), which promotes NHEJ-c while inhibiting HR [27,28]. After DSB signaling, the HR activates the Mre11/Rad50/NSB1 complex, end-resection, and the stabilization of DNA strands by the RPA protein, which is then replaced by RAD51 [29]. Several studies have shown that hypoxia increases the phosphorylation of ATM and DNA-PKcs [30,31,32], modulates γH2AX [33] or downregulates HR [29,31]. Cam et al. showed that the ATM protein was able to stabilize HIF-1α by inducing its phosphorylation at S696 [34]. Furthermore, γH2AX induction was delayed in HIF-1α deficient MEF cells and decreased in HEK293T cells after HIF-1α knockdown [35]. However, if the initiation and detection of DSBs seem to be modulated by HIF-1α, the consequences on the downstream pathways, i.e., NHEJ-c and HR, have not been investigated. Our work aims to determine the contribution of HIF-1α under hypoxia on the DSBs repair after photon or C-ion exposure, from the DSBs detection to the activation of the HR and/or NHEJ-c, in two HNSCC cell lines and their CSC subpopulation.

## 2. Materials and Methods

### 2.1. Cell Culture

Two HNSCC radioresistant cell lines, SQ20B (RRID: CVCL_7138) and FaDu (RRID: CVCL_1218) were established from HNSCC tumors (larynx and pharynx), and respectively provided by J.B. Little (Department of Cancer Biology, Harvard School of Public Health, Boston, MA, USA) and purchased from the American Type Culture Collection (Manassas, VA, USA). Their sub-population of non-CSCs (FaDu^CD44Low^) and CSCs (SQ20B-CSCs and FaDu-CSCs) were obtained by flow-cytometry cell sorting and cultured as described in [16,20,36]. The parental SQ20B cell line, which contains less than 1% of CD44-positive cells, was defined as the non-CSC control cells for SQ20B-CSCs and was named SQ20B^CD44low^.

### 2.2. Hypoxic Conditions

Cells were cultured under normoxia (21% O_2_) or hypoxia (1% O_2_) in a tri-gas chamber (Heracell 150i, Thermo Fisher Scientific, Waltham, MA, USA; or 9000E; Wakenyaku, Kyoto, Japan). For hypoxic conditions, cells were grown in hypoxia for 20–24 h before irradiation. During irradiation, Lab-Teks^TM^ (Dutscher, Bruxelles, Belgium) were covered with an airtight plastic film to limit oxygen exchanges and were immediately replaced after exposure in a hypoxic incubator for kinetic studies (30 min to 24 h).

### 2.3. Irradiations

Cells were irradiated at a dose rate of approximately 2 Gy/min with 250 kV photons (X-RAD320, Lyon-Sud Medical School, Lyon, France) and C-ions at the initial energy of 290 MeV/n, center of 6 cm Spread-Out Bragg Peak (SOBP) with the Heavy-Ions Medical Accelerator of Chiba, at National Institute of Radiological Sciences (Chiba, Japan) [16,20,37]. Time points were performed from 30 min after irradiation (time needed to eliminate neutrons in the irradiation room and come back to the bench) to 24 h. The radiobiological parameters (α, β, RBE at 10% survival) calculated according to the linear-quadratic model were previously published for the four subpopulations studied [16,38]. Following a comparative study for DNA Damage Repair kinetics between X-rays and C-ions, and since RBEs were dependent on both the populations tested and the oxygen concentration, all experiments were performed at an equivalent physical dose of 2 Gy photons [38]. Institutional safety was approved and followed for culture of human cell lines.

### 2.4. Transient Transfections

Cells were transfected with two siRNA targeting HIF-1α (exon 5 (#42840) or exon 12 (#s6539), respectively) as well as an irrelevant siRNA (Thermo Fisher Scientific, Carlsbad, CA, USA) as previously described and validated [16].

### 2.5. Immunofluorescence

Cells were seeded the day before experiments on Lab-Teks^TM^ (Dutscher) at a density of 3.10^5^ cells per slide and incubated at 37 °C under normoxia or hypoxia before irradiation. After radiation exposure (30 min to 24 h), cells were fixed for 15 min in 4% PFA, permeabilized, and labeled according to recommendations of the manufacturer for each primary antibody, i.e., 1 h at room temperature (1:1000, mouse anti-phospho-histone H2AX-Ser139 (Merck, Kenilworth, NJ, USA); 1:750 rabbit anti-Rad51 (Abcam, Cambridge, GB); 1:250, rabbit anti-53BP1 (NovusBio, Littleton, CO, USA); 1:250, rabbit anti-phospho-ATM-S1981 (Abcam); 1:50 mouse anti-HIF-1α (BD Transduction, San-José, CA, USA), and 1:500, rabbit anti-phospho-DNA-PKcs-S2056 (Abcam)) and secondary antibodies, i.e., for 1 h in the dark at room temperature (1:500, anti-IgG rabbit Alexa Fluor 488 (Invitrogen, Carlsbad, CA, USA); 1:250, anti-IgG mouse Alexa Fluor 555 (Abcam)). Nuclei were stained for 15 min with 1 µg/mL DAPI (Sigma, Kanagawa, Japan), then slides were mounted using Fluoromount (Merck) and stored at room temperature in the dark until analysis [38] (*n* = 2).

### 2.6. Microscopy

The fluorescent signals of 300 nuclei were quantified at 63× in duplicate with the automated-reading platform Metafer (MetaSystems GmbH, Altlußheim, Germany), which is composed of a motorized optical microscope (Axio Imager.Z2; Carl Zeiss, Germany) with a monochromatic camera (CCD CoolCubeR 1m; MetaSystems GmbH) and a 200 V dimmable mercury lamp (X-citeR; Excelitas Technologies, Waltham, MA, USA) (*n* = 2). The numbers of foci per nucleus were quantified for P-ATM, RAD51, γH2Ax, P-DNA-PK, and 53BP1 after automatic delimitation of the nucleus according to DAPI fluorescence. For kinetic studies, the peaks of photons were defined as the reference (100%) and normalized in each condition to this peak in order to compare the variations of signals as a result of the expression of HIF-1α. The percentages of foci per nucleus were calculated for each condition, and the means ± SD are presented in each figure. The speed of the peak induction was determined from basal levels to the peak values and the decay-rate of the foci was calculated from the number of foci at peak to half of the peak value in foci.min^−1^.

### 2.7. Protein Studies by Western-Blot

Cells transfected with a siRNA targeting HIF-1α or an irrelevant siRNA were irradiated or not with 2 Gy photons or C-ions, and then incubated under normoxic or hypoxic conditions. After 0.5 h to 24 h post-irradiation, cells were lysed, and the protein concentration was determined [17]. Proteins were separated on 7% polyacrylamide gels, and transferred onto a nitrocellulose membrane. The lower part of the gel containing actin (42 kDa) was transferred with a Trans-Blot Turbo Transfer System (Biorad, Hercules, CA, USA), whereas the upper part containing the proteins of interest (respectively 350 kDa for P-ATM and ATM, and 460 kDa for P-DNA-PKcs and DNA-PKcs) was submitted to a 16 h liquid transfer. The antibodies used for blotting were rabbit anti-P-ATM (Abcam 81292; 1:50,000); rabbit anti-ATM (Cell signaling 2873; 1:1000); rabbit anti-P-DNA-PKcs (Cell signaling 2873; 1:1000); anti-P-DNA-PKcs (Abcam 18192; 1:400); rabbit anti-DNA-PKcs (Abcam 32566; 1:1000); and mouse anti-actin (Sigma; 1:50,000). Secondary antibodies used were respectively HRP-conjugated anti-rabbit and mouse IgG (both 1:5000; Cell signaling 7074 and 7076). Phosphorylated proteins were firstly analyzed and the nitrocellulose membrane was then incubated for 10 min with the Western Blot stripping buffer (Thermo Scientific, Rockford, IL, USA). Non-phosphorylated signals (ATM and DNA-PKcs) were then analyzed. Signals were measured by densitometric scanning with an Azure C300 Intelligent Dark Box (Biosystems Inc., Dublin, CA, USA), and protein expressions were quantified with MultiGauge (FujiFilm, Tokyo, Japan) after actin normalization (Figures S1 and S2).

### 2.8. Statistical Analysis

Two-way ANOVA multiple comparison tests or Student’s *t*-tests were applied using GraphPad Prism 8.4.2 (San Diego, CA, USA) and Excel software, to assess the significance of differences between two groups, with correction for multiple comparisons relying on the Holm–Sidak method. *p*-values ≤ 0.05 were considered statistically significant, and the levels of significance indicated as follows (**** *p*< 0.0001, *** *p*< 0.001, ** *p*< 0.01, and * *p* < 0.05).

## 3. Results

### 3.1. HIF-1α Nucleoshuttling in Response to X-ray and C-Ion Irradiation

**Figure 1:**
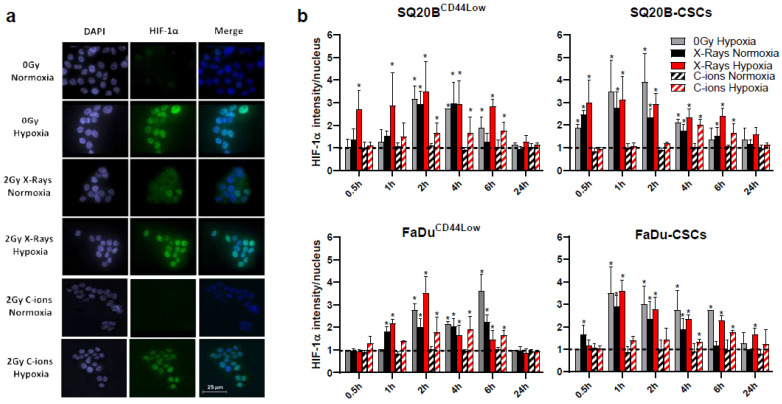
HIF-1α nucleoshuttling is induced by photons under normoxia and hypoxia, but only under hypoxia with C-ions (**a**) Representative images at 4 h of SQ20B^CD44Low^ cells (0 or 2 Gy photons and C-ions, under normoxia or hypoxia) are presented with the indicated biomarkers. (**b**) Signal intensities of HIF-1α into the nucleus were measured for each condition and normalized to basal signal. The means ± SD were statistically compared to the basal ratio. (Student’s *t*-Tests using Holm–Sidak method, α = 0.5). (*n* = 3 photons; *n* = 2 C-ions). * *p* < 0.05.

After HIF-1α stabilization in the cytoplasm following hypoxia, this subunit is expected to translocate to the nucleus and dimerize with HIF-1β in order to act as an active transcriptional factor. We therefore investigated this translocation from the cytoplasm to the nucleus, called nucleoshuttling, by immunofluorescence experiments in the four cell populations: SQ20B^CD44Low^, FaDu^CD44Low^, SQ20B-CSCs, and FaDu-CSCs (Figure 1).

Representative images at 4 h of SQ20B^CD44Low^ labeled with HIF-1α antibody and DAPI are shown in Figure 1a. The means of the nucleus signal intensities normalized to respective basal signals are presented in Figure 1b. Under normoxic conditions, the nucleoshuttling of HIF-1α was induced by X-rays, at an earlier time in CSCs (maximum at 1 h) compared to non-CSCs (between 2 h and 6 h), whereas C-ions did not activate this translocation. Under hypoxia, the nucleoshuttling of HIF-1α was enhanced after both photon and C-ion exposure, with a maximum delayed (between 4 h and 6 h) for C-ions. Then, the potential interrelation of HIF-1α with the DSBs repair pathways was investigated under hypoxia, considering the normoxic condition as the reference.

### 3.2. Silencing HIF-1α under Hypoxia Decreases the Initiation of the Signaling of DSBs after Photon or C-Ion Exposure

**Figure 2:**
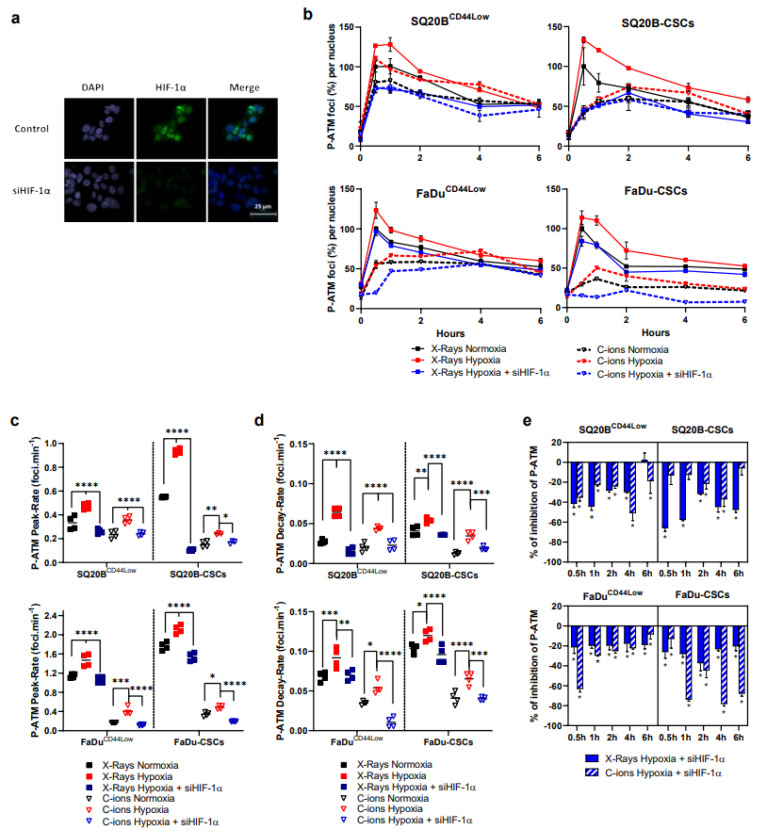
The silencing of HIF-1α combined with photons and C-ions decreases the initiation of the signaling of DSBs under hypoxia. (**a**) Representative images of silencing of HIF-1α in SQ20B^CD44Low^ cells under hypoxia at 4 h. (**b**) Kinetics of ATM phosphorylation (P-ATM) after 2 Gy X-rays or C-ions, ± siHIF-1α. The percentages of foci per nucleus were calculated for each condition considering the X-ray normoxic peak as the reference. The symbols and error bars indicate means ± SD values. (**c**,**d**) represent respectively the P-ATM peak-rate from basal levels to peak and its decay-rate from peak to 6 h, as determined from the data shown in (**b**). Each plot represents the mean ± SD values (two-way ANOVA test, * *p* < 0.05, ** *p* < 0.01, *** *p* < 0.001, **** *p* < 0.0001). (**e**) Effect of HIF-1α silencing on p-ATM expression under hypoxia. The p-ATM percentages between hypoxic conditions were statistically compared (Student’s *t*-Tests, Holm–Sidak method, α = 0.5) (*n* = 3 photons; *n* = 2 C-ions).

First, the expression of the total amount of the ATM protein and its phosphorylated form was studied by Western-blot after 2 Gy photons or C-ions after HIF-1α silencing or not, and under normoxic or hypoxic conditions in SQ20B^CD44Low^, SQ20B-CSCs, FaDu^CD44Low^, and FaDu-CSCs (Figure S1). The quantifications of the expression of ATM normalized with actin show that the recruitment of the ATM protein did not change in the four populations under the different conditions, whereas a variation of the phosphorylated ATM (P-ATM) was observed (Figure S2). Since Western-blots are unable to give information on the intra-cellular localization of the phosphorylated form of ATM, and as only P-ATM present in the nucleus has a role in the repair, the effects of HIF-1α silencing on the phosphorylation of ATM were studied after 2 Gy X-rays or C-ions by fluorescence microscopy. To specifically study the DNA-repair process, the number of foci induced by the different conditions was counted in the nucleus (Figure 2). 

Representative images for SQ20B^CD44Low^ cells are shown in Figure 2a, which assesses the efficacy of HIF-1α silencing. After X-rays, no difference was observed in the phosphorylation of ATM under normoxia whether after HIF-1α silencing or not (Figure S3). For all the other experimental conditions used, the percentages of foci per nucleus were calculated for each condition considering the normoxic X-ray peak as the reference (Figure 2b). The four kinetics obtained show a significant increase of P-ATM signals under hypoxia compared with normoxia, while HIF-1α silencing decreases the P-ATM signals after both types of radiation. Representative images of foci per nucleus are shown in Figure S4 for each cell line in the basal condition, at peak and 6 h.

As shown in Figure 2c,d, which respectively represent the rate of induction of the P-ATM peak and its decay-rate after both radiations, the peaks were reached faster under hypoxia and the decay-rates accelerated. However, silencing HIF-1α combined with X-ray or C-ion exposure under hypoxia efficiently decreased both the speed of the peak induction and its decay in both populations, which suggests a lower initiation of the DSB signaling in these conditions. Furthermore, the decrease of P-ATM signals was confirmed after silencing HIF-1α under hypoxia (Figure 2e). Altogether, our results show that silencing HIF-1α under hypoxia significantly decreases the initiation of the DSB signaling after both photons and C-ions in non-CSCs and CSCs subpopulations.

### 3.3. Silencing HIF-1α Modulates the Detection of DSBs in Response to Both Exposure, Particularly by Increasing Residual γH2AX Foci

**Figure 3:**
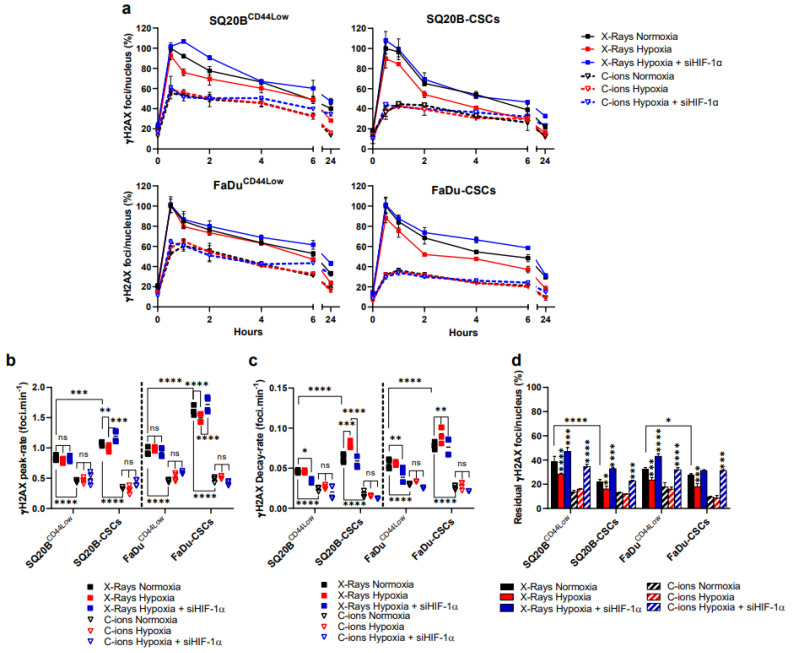
Silencing HIF-1α under hypoxia increases the residual detected DSBs in response to both irradiations. (**a**) Kinetics of γH2AX foci after X-rays and C-ions, ± siHIF-1α. The percentages of foci per nucleus were calculated for each condition considering the X-ray normoxic peak as the reference (means ± SD). (**b**,**c**) represent respectively the peak-rate from basal levels and decay-rate from peak to 6 h, as determined from the data shown in (**a**) (two-way ANOVA test, * *p* < 0.05, ** *p* < 0.01, *** *p* < 0.001, **** *p* < 0.0001, ns: non-significant). (**d**) Residual foci were quantified at 24 h and each condition was statically compared to their respective normoxic condition (two-way ANOVA test) (*n* = 3).

The potential involvement of HIF-1α in the detection of DSBs induced by photons and C-ions was investigated through the kinetics of phosphorylation of H2AX (γH2AX) in non-CSCs and CSC HNSCCs cell lines (Figure 3a). Representative images of foci per nucleus are shown in Figure S5 for each cell line in the basal condition, at peak and 24 h.

After photon exposure, the γH2AX signals were decreased under hypoxia in the four subpopulations tested. Moreover, the initial peak-rate was reached more slowly in CSCs under hypoxia whereas the decay-rate was accelerated. This was not observed in response to C-ions. After HIF-1α silencing following hypoxia and photon exposure, no change occurred concerning the peak rate of γH2AX in non-CSCs (Figure 3b) but a decreased decay and an enhancement of residual γH2AX foci were observed (Figure 3c,d). By contrast, an accelerated peak-rate and a decrease of the decay-rate in CSCs were observed. Following C-ion exposure, the silencing of HIF-1α under hypoxia had no influence on the peak-rate and decay of γH2AX, whatever the subpopulation considered but resulted in an enhancement of residual γH2AX foci (Figure 3d). Altogether, these results underline the predominant role of HIF-1α on the detection of DSBs in CSCs, particularly after photon exposure. Considering the residual γH2AX foci, our results suggest that a better repair of the DSBs occurred after photon exposure compared with C-ions, without any effect of the oxygen concentration with this latter. After HIF-1α silencing following hypoxia, the enhancement of residual foci was obtained whatever the experimental conditions used. This confirms the involvement of HIF-1α in the detection of DSBs after both exposure and thus a likely less efficient DNA repair.

### 3.4. 53BP1 Is Not Significantly Modulated by HIF-1α Expression

**Figure 4:**
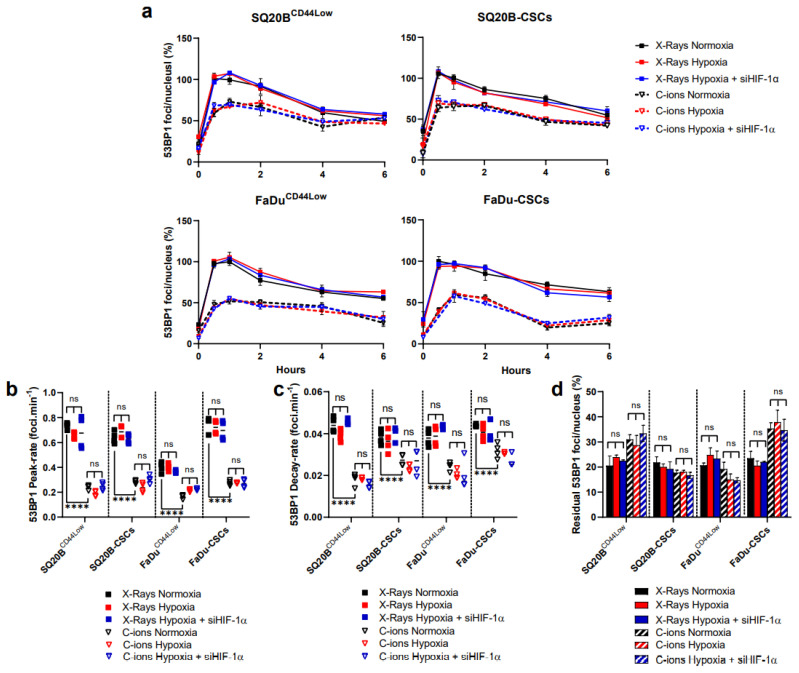
HIF-1α silencing does not impact 53BP1 levels of foci. (**a**) Kinetics of 53BP1 foci after X-rays and C-ions ± siHIF-1α. The percentages of foci per nucleus were calculated for each condition considering the X-ray normoxic peak as the reference (means ± SD). (**b**,**c**) represent respectively the 53BP1 peak-induction from the basal levels and the decay-rate from the peak to 6 h, as determined from the data shown in (**a**). (**d**) Residual foci were quantified at 24 h and each condition was compared to their respective normoxic condition (two-way ANOVA test, ns = non-significant). (*n* = 3 photons; *n* = 2 C-ions).

Tumor suppressor p53-binding protein (53BP1) is known to play a key role in the balance between the two main DSBs repair pathways, i.e., the NHEJ-c and the HR pathways. The contribution of HIF-1α to the choice of the DNA repair pathway was therefore investigated following both photon and C-ion exposure (Figure 4). Representative images of foci per nucleus are shown in Figure S6 for each cell line in the basal condition, at peak and 6 h.

After photon exposure, a higher activation of 53BP1 was observed compared with C-ions in the four populations studied. However, following hypoxia, the 53BP1 kinetics (Figure 4a), the rates of the induction and decay of the peak (Figure 4b,c), as well as the residual foci at 24 h (Figure 4d), were not modified regardless of the radiation applied and the cell subpopulations used in these experiments. Moreover, the silencing of HIF-1α did not influence the recruitment of 53BP1. These results suggest that under our experimental conditions, HIF-1α is unable to significantly turn toward a preferential DSB repair pathway.

### 3.5. Silencing HIF-1α Decreases the NHEJ-c Pathway through DNA-PKcs in Response to Photons and C-Ions

**Figure 5:**
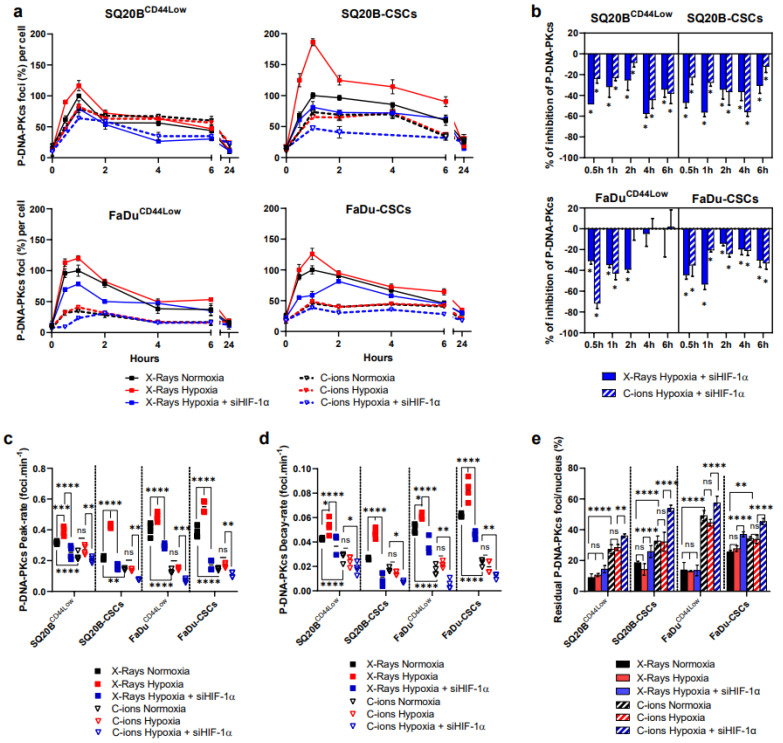
HIF-1α silencing under hypoxia decreases the NHEJ-c pathway after both irradiations. (**a**) Kinetics of P-DNA-PKcs foci after X-rays and C-ions ± siHIF-1α. The percentages of foci per nucleus were calculated for each condition considering the X-ray normoxic peak as the reference (means ± SD). (**b**) Effect of HIF-1α silencing under hypoxia on P-DNA-PKcs expression. The percentages between hypoxia ± siHIF-1α were statistically compared (Student’s t-Tests, Holm–Sidak method, α = 0.5) (*n* = 3 photons; *n* = 2 C-ions). (**c**,**d**) represent respectively the induction-rate of the P-DNA-PKcs peak from the basal levels, and its decay-rate from the peak to 6 h, as determined from the data shown in (**a**) (two-way ANOVA test, * *p* < 0.05, ** *p* < 0.01, *** *p* < 0.001, **** *p* < 0.0001, ns: non-significant). (**e**) Residual foci were quantified at 24 h and each condition was compared to their respective normoxic condition (two-way ANOVA test) (*n* = 3 photons, *n* = 2 C-ions).

We next investigated DNA-PKcs as a specific marker of the NHEJ-c repair pathway downstream of the initiation and signaling described above. As well as for ATM, the expressions of the total amount of the DNA-PKcs protein and its phosphorylated form were studied (Figure S1). The quantifications of the expression of DNA-PKcs normalized to actin show that there is no modification of its expression in the four populations, whatever the conditions used (Figure S2). Then, the effects of HIF-1α silencing on the phosphorylation of DNA-PKcs (P-DNA-PKcs) were studied by microscopy after 2 Gy X-rays or C-ions (Figure 5). 

Representative images of foci per nucleus are shown in Figure S7 for each cell line in the basal condition, at peak and 24 h.

Following hypoxia and photon exposure, a faster induction and decrease of the P-DNA-PKcs peak compared to normoxia (Figure 5c,d) occurred in the four subpopulations without any significant effect on the number of residual foci. By contrast, these effects were not observed in response to C-ions. Silencing HIF-1α under hypoxia however decreased the peak rate and the decay signals, as well as the kinetics of P-DNA-PKcs at most of the time points, after both exposure and whatever subpopulation was studied.

Additionally, the residual P-DNA-PKcs foci were quantified 24 h after both irradiations (Figure 5e). The results obtained show that the silencing of HIF-1α under hypoxia resulted in an increase of residual P-DNA-PKcs foci only in CSCs after photon exposure, whereas this increase was obtained at a higher proportion in non-CSCs and CSCs after C-ion exposure. Altogether, our results present evidence that HIF-1α is involved in the modulation of the NHEJ-c pathway since its silencing decreases the kinetics of P-DNA-PKcs after both exposure, which is associated with higher residual foci especially after C-ion exposure.

### 3.6. Silencing HIF-1α Decreases HR in Response to Photon and C-Ion Exposure

**Figure 6:**
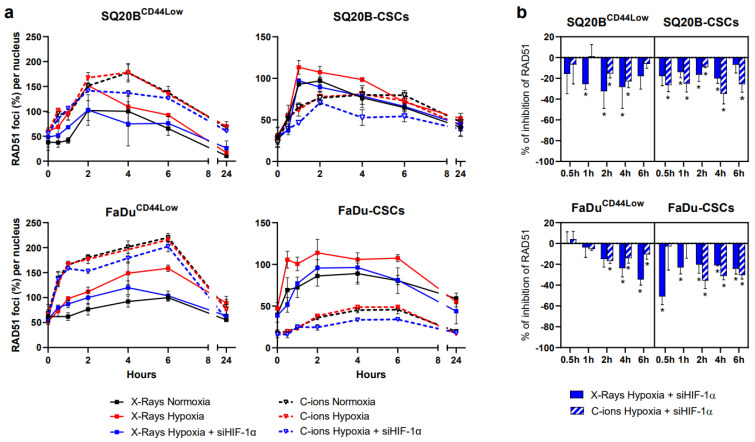
HIF-1α inhibition under hypoxia decreases HR in response to photons and C-ions. (**a**) Kinetics of RAD51 foci after X-rays and C-ions HIF-1α ± siHIF-1α. The percentages of foci per nucleus were reported to the reference (X-rays-Normoxia) and calculated for each condition (means ± SD). (**b**) Effect of the silencing of HIF-1α under hypoxia on RAD51 expression. The RAD51 percentages between hypoxia ± siHIF-1α were statistically compared (Student’s *t*-Tests, Holm–Sidak method, α = 0.5) (*n* = 3). * *p* < 0.05.

Since RAD51 is a prominent marker of the HR repair pathway, its kinetics of expression following HIF-1α inhibition were analyzed after both irradiations (Figure 6a). Representative images of foci per nucleus are shown in Figure S8 for each cell line in the basal condition, at 2 h and 24 h.

Following hypoxia, the kinetics of RAD51 foci were enhanced after photon exposure in all the subpopulations studied. By contrast, no modification occurred after C-ion exposure, a result in accordance with our previous studies [38]. Interestingly, silencing HIF-1α after hypoxia induced a strong decrease of RAD51 in response to both radiations in non-CSCs and CSCs, as shown in Figure 6b. Under hypoxia, these results suggest a lesser activation of HR after HIF-1α silencing independently of the radiation applied.

## 4. Discussion

Hypoxia plays a central role in HIF-1α stabilization and induces various changes in the expression and activity of genes involved in DSB repair. Since HIF-1α expression is associated with worse outcomes [15], many chemical compounds and drugs have been tested, showing inhibition of HIF-1α and subsequent processes involved such as tumor growth or metastasis [8,39]. The impressive number of inhibitors that have been studied reflects the huge interest in targeting HIF-1α in cancer therapy and the significance of identifying and characterizing its impact in relative cellular pathways [40]. Thus, silencing HIF-1α combined with photon and C-ion irradiations induces HNSCC cell sensitization under hypoxia [16,20], suggesting its involvement in the modulation of the DNA Damage Response (DDR).

This study clearly highlights the involvement of HIF-1α in the detection, signaling, and repair of the DNA Double-Strand-Breaks in response to both irradiations under hypoxia, in two HNSCC cell lines and their subpopulation of Cancer-Stem Cells. Here, our experiments demonstrate the interrelation of HIF-1α with the DDR after photon and C-ion exposure through a decreased initiation of the detection of DSBs and increased signaling of DSBs, but also a decrease of P-DNA-PKcs (NHEJ-c) and RAD51 (HR) signals, without favoring one of them through 53BP1 expression.

First, the nucleoshuttling of HIF-1α was effective under normoxia only in response to photons, whereas this stabilization was effective after photons and C-ions under chronic hypoxia, thus confirming previous data obtained under acute hypoxia [16].

Our results further underline the contribution of HIF-1α in the initiation of the detection and the signaling of DSBs in response to both irradiations under hypoxia. Suppressing HIF-1α expression under chronic hypoxia in combination with photons and C-ions decreased the initiation of the detection of the DSBs in non-CSCs and CSCs, but it also increased the γH2AX residual foci. After chemical induction of DSBs, Wrann *et al.* showed a delayed decrease in γH2AX foci induction after HIF-1α silencing under severe hypoxia in cancer cell lines, suggesting an interaction between HIF-1α and γH2AX [35]. Additionally, according to Lin’s group, the phosphorylated-histone H2AX triggered by ATM during hypoxia interacts with HIF-1α to maintain its stability and nuclear accumulation, thereby inducing the activation of signaling pathways involved in the cellular response to hypoxia [41]. They also showed that this interaction is dependent on TRAF6 and mono-ubiquitination, which preserves HIF-1α from degradation. Because of our results showing that HIF-1α interplays with the initiation of the detection and the signaling of the DSBs in hypoxic conditions after photons and C-ions, we then expected potential consequences on the downstream repair pathways.

If Harding et al. previously showed that 53BP1 is activated specifically in S-phase cells during anoxia in an ATM-dependent manner in colorectal cancer cells [42], our results do not support a significant modulation of this marker or the HR by hypoxia and HIF-1α in HNSCC cells [42]. Depending on the degree of hypoxia (severe or mild, acute, or chronic), previously published studies globally reported an increase in the phosphorylation of DNA-PKcs [30,32]. In our work, silencing HIF-1α with both irradiations efficiently downregulated the NHEJ-c and the HR in HNSCC cell lines. Interestingly, C-ions were particularly efficient on HR compared with photons in CSCs, leading to a worse repair of the DSBs and explaining in part the better efficacy of C-ions on CSCs compared with photons. Some other studies have on the other hand demonstrated a decrease of RAD51 expression under mild hypoxia, in a cell cycle and HIF-1-independent manner [29]. However, it has also been reported that cells under severe hypoxia show different responses from those under mild hypoxia, where oxygen concentration is 0.5–2.0% [32]. Many genes involved in the DDR have been identified as dependent on HIF-1α regulation. Even if DDR protein levels were increased by hypoxia in a HIF-1α-dependent manner, Calvo-Asensio et al. showed that hypoxia did not modify mRNA levels of DDR genes such as DNA-PKcs [43]_._ Our data, combined with that of the literature confirm an interrelation of HIF-1α with the detection, signaling, and repair pathways of DSBs. Additionally, they more likely suggest that HIF-1α, through the transcriptional regulation of intermediate proteins, may indirectly affect DDR protein stability.

Overall, it appears that targeting HIF-1α may be a useful therapeutic approach to decrease DRR in response to radiotherapy. Since we previously showed the role of HIF-1α in the radioresistance of HNSCC, the development of selective HIF-1α inhibitors is then required to specifically and robustly block its expression. Recently, promising strategies have been proposed, relying on the CRISPR-CAS9 approach, novel nano-based drug delivery systems, which increased the detection of HIF factors at tumor sites, and HIF-1α-specific siRNA-loaded nanoparticles, which inhibit tumor metastasis in pancreatic models, allowing a better and more specific tumor delivery [40,44,45].

## 5. Conclusions

Collectively, our findings demonstrate the interrelation of HIF-1α with DSB repair pathways under chronic hypoxia in HNSCC non-CSCs and CSCs after both photon and C-ion irradiations, thus highlighting HIF-1α as an important target to awaken radioresistance and the need to develop specific inhibitors. Silencing HIF-1α in association with irradiation thus constitutes a promising strategy to target radioresistant tumor cells in hypoxic niches.

## Data Availability

The datasets generated during and/or analyzed during the current study are available from the corresponding author on reasonable request.

## Supplementary Materials

The following are available online at https://www.mdpi.com/article/10.3390/cancers13153833/s1, Figure S1: Expression of ATM, P-ATM, DNA-PKcs and P-DNA-PKcs by Western-blot from 30 min to 24 h after 2 Gy X-rays or C-ions ± siHIF-1α under normoxic and hypoxic conditions. Figure S2: Quantification of the expression of ATM. Figure S3: Kinetics of ATM phosphorylation (P-ATM) after 2 Gy X-rays ± siHIF-1α. Figure S4: Representative images of P-ATM foci per nucleus in SQ20B^CD44Low^, FaDu^CD44Low^, SQ20B-CSCs and FaDu-CSCs. Figure S5: Representative images of γH2AX foci per nucleus in SQ20B^CD44Low^, FaDu^CD44Low^, SQ20B-CSCs and FaDu-CSCs. Figure S6: Representative images of 53BP1 foci per nucleus in SQ20B^CD44Low^, FaDu^CD44Low^, SQ20B-CSCs and FaDu-CSCs. Figure S7: Representative images of P-DNA-PKcs foci per nucleus in SQ20B^CD44Low^, FaDu^CD44Low^, SQ20B-CSCs and FaDu-CSCs. Figure S8: Representative images of RAD51 foci per nucleus in SQ20B^CD44Low^, Fa-Du^CD44Low^, SQ20B-CSCs and FaDu-CSCs.
