# Supplementary material for: Involvement of HIF-1α in the Detection, Signaling, and Repair of DNA Double-Strand Breaks after Photon and Carbon-Ion Irradiation
Source: Cancers (Basel). 2021 Jul 30;13(15):3833. doi: 10.3390/cancers13153833 (PMC8345054; doi:10.3390/cancers13153833)
Supplement: Supplementary file 1 [file cancers-13-03833-s001.zip › Supplementary figures proof.pdf]

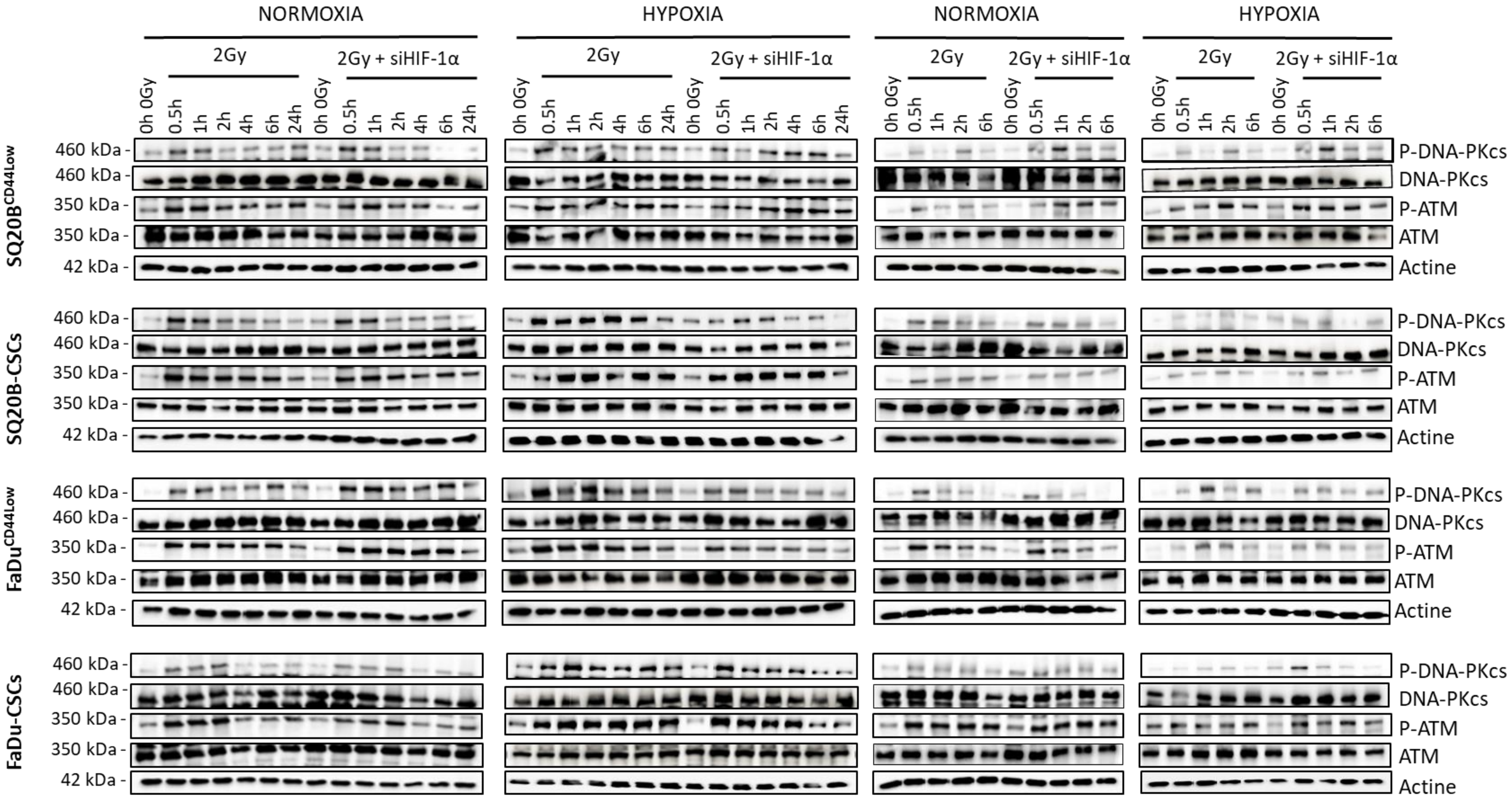

Supplementary Figure S1: *Expression of ATM, P-ATM, DNA-PKcs and P-DNA-PKcs by Western-blot from 30 min to 24 h after 2 Gy X-Rays or C-ions ± siHIF-1α under normoxic and hypoxic conditions. (n=1)*

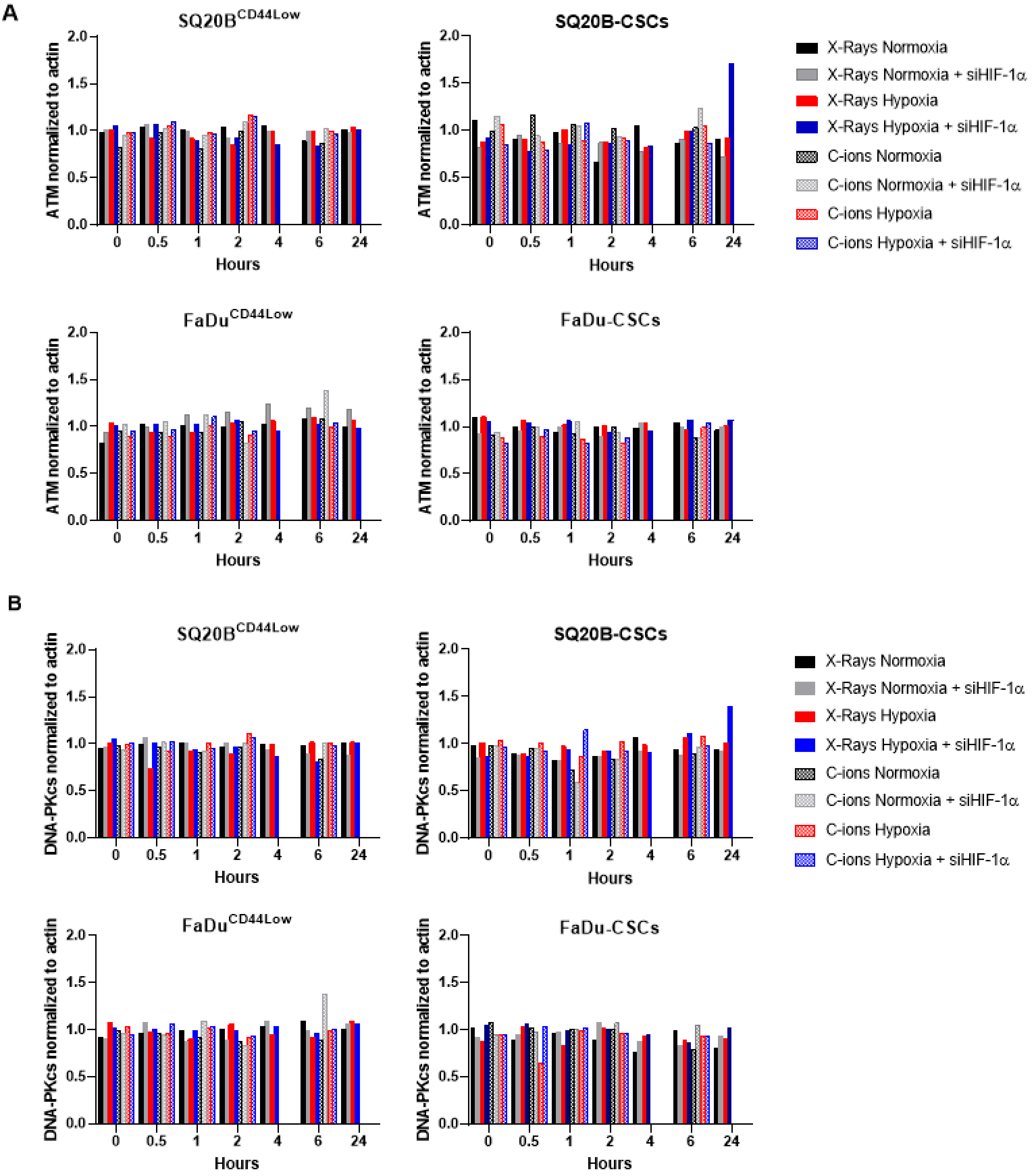

Supplementary Figure S2*: Quantification of the expression of ATM (A) and DNA-PKcs (B) normalized to actin obtained by Western-blot in Supplementary Figure 1.*

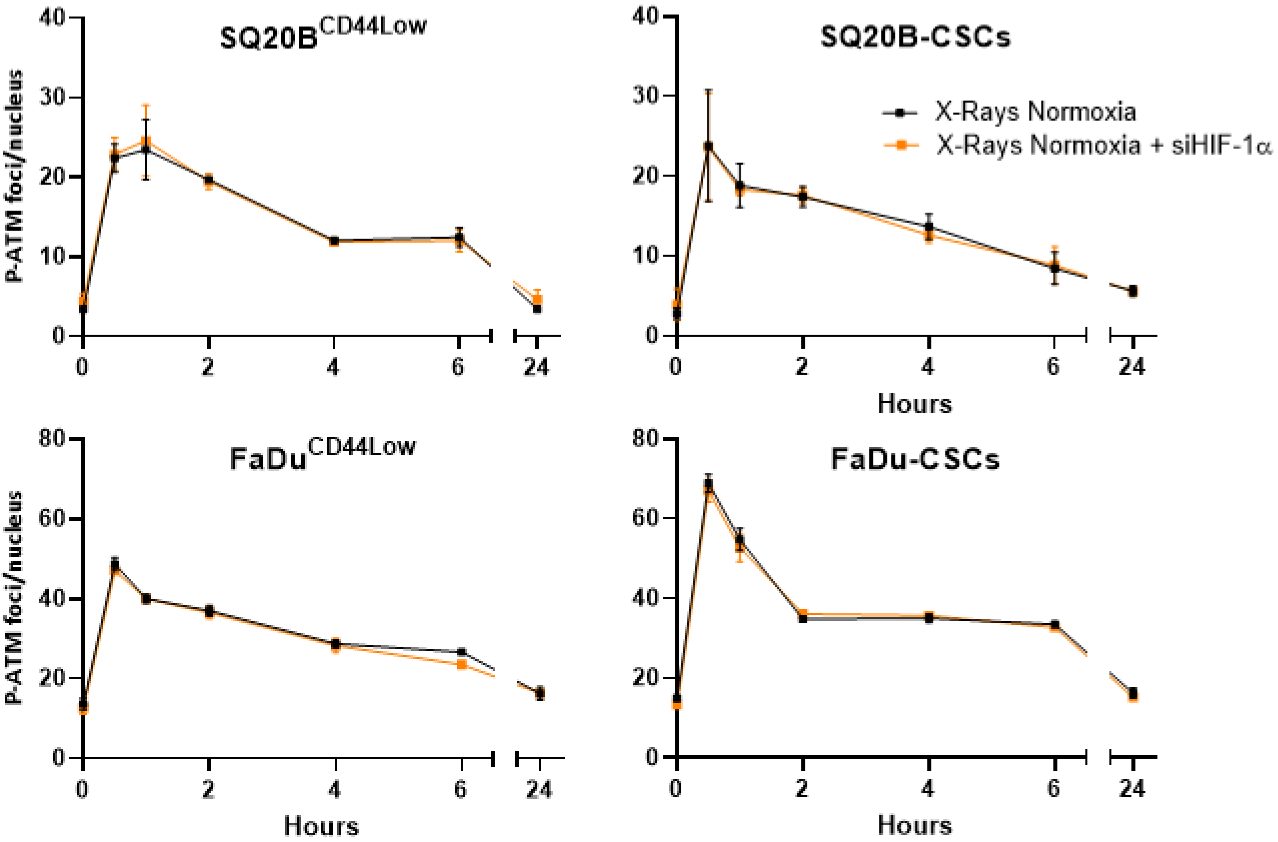

Supplementary Figure S3*: Kinetics of ATM phosphorylation (P-ATM) after 2 Gy X-Rays ± siHIF-1α. The symbols and error bars indicate means ± SD values (Student's t-Tests, Holm-Sidak method, α=0.5).*

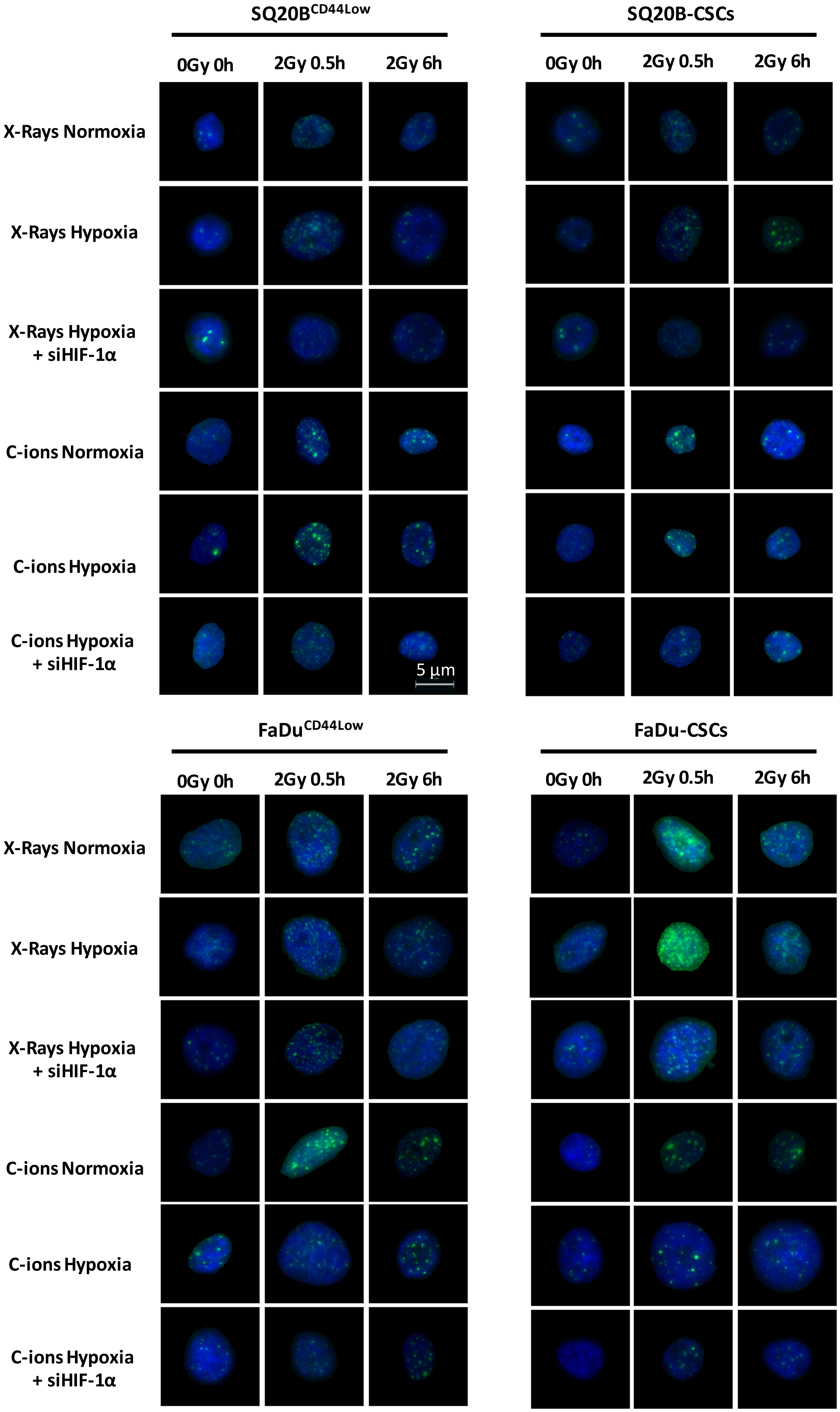

Supplementary Figure S4: *Representative images of P-ATM foci per nucleus in SQ20B^{CD44Low}, FaDu^{CD44Low}, SQ20B-CSCs and FaDu-CSCs.*

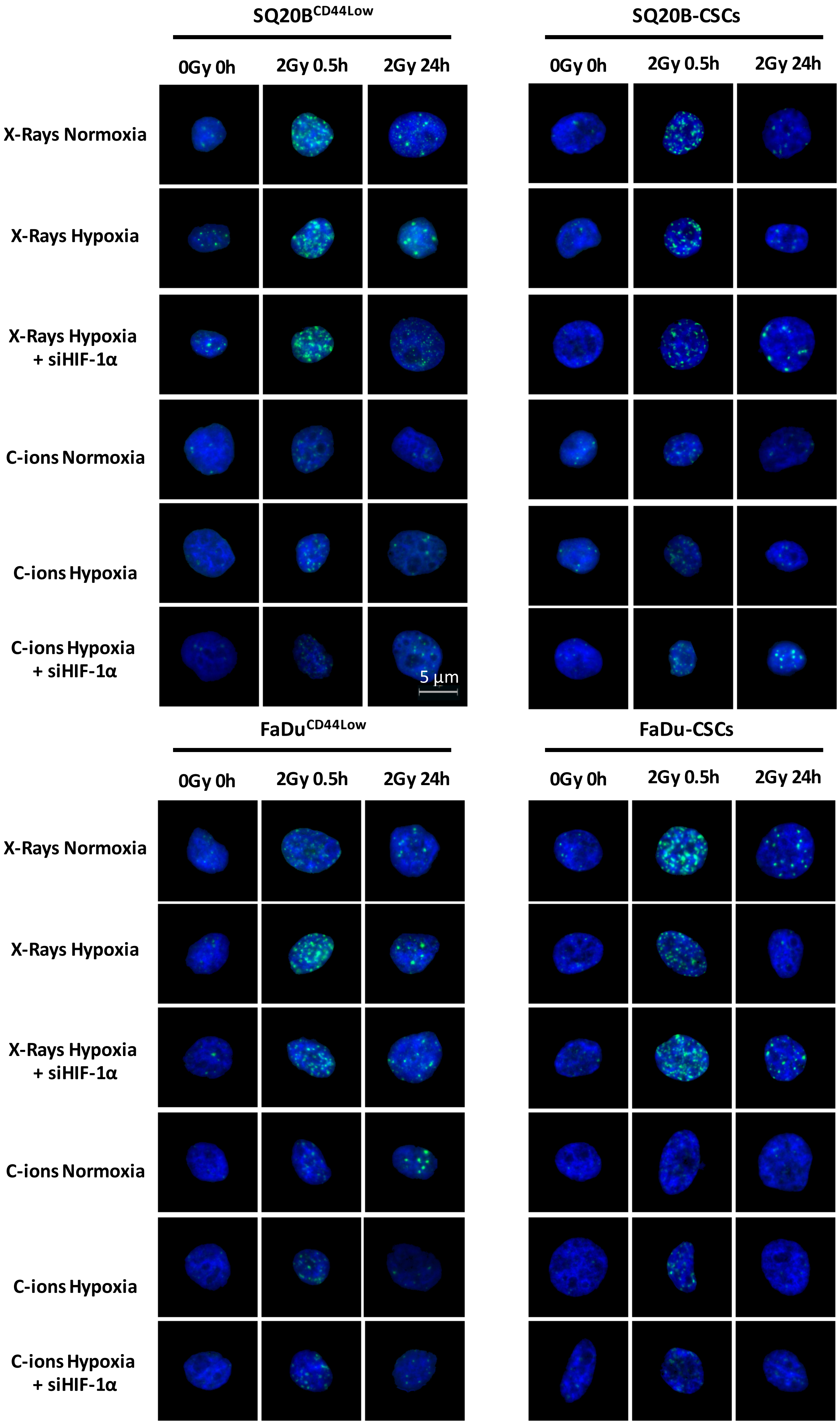

Supplementary Figure S5: *Representative images of γH2AX foci per nucleus in SQ20B^{CD44Low}, FaDu^{CD44Low}, SQ20B-CSCs and FaDu-CSCs.*

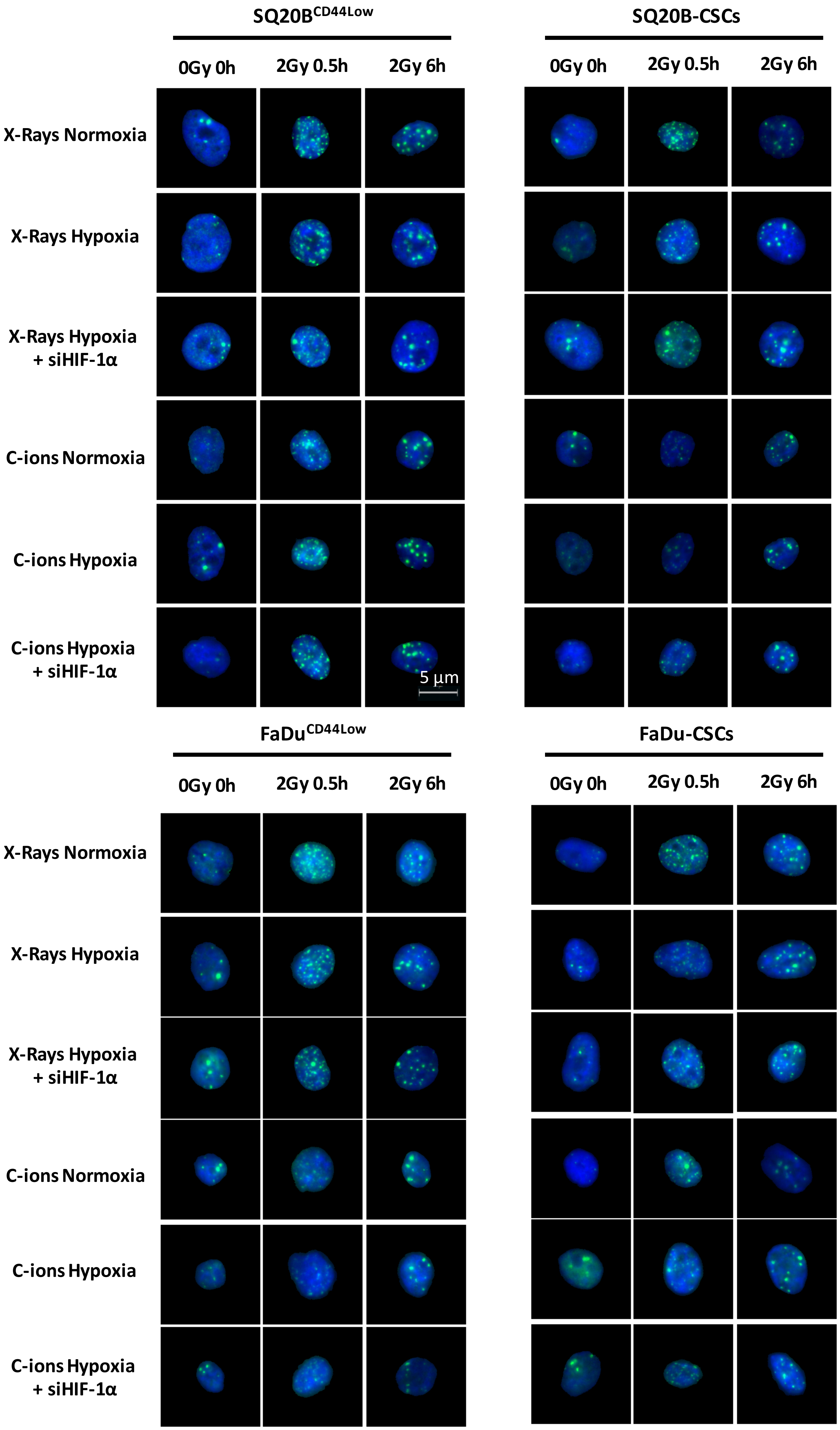

Supplementary Figure S6*: Representative images of 53BP1 foci per nucleus in SQ20B^{CD44Low}, FaDu^{CD44Low}, SQ20B-CSCs and FaDu-CSCs.*

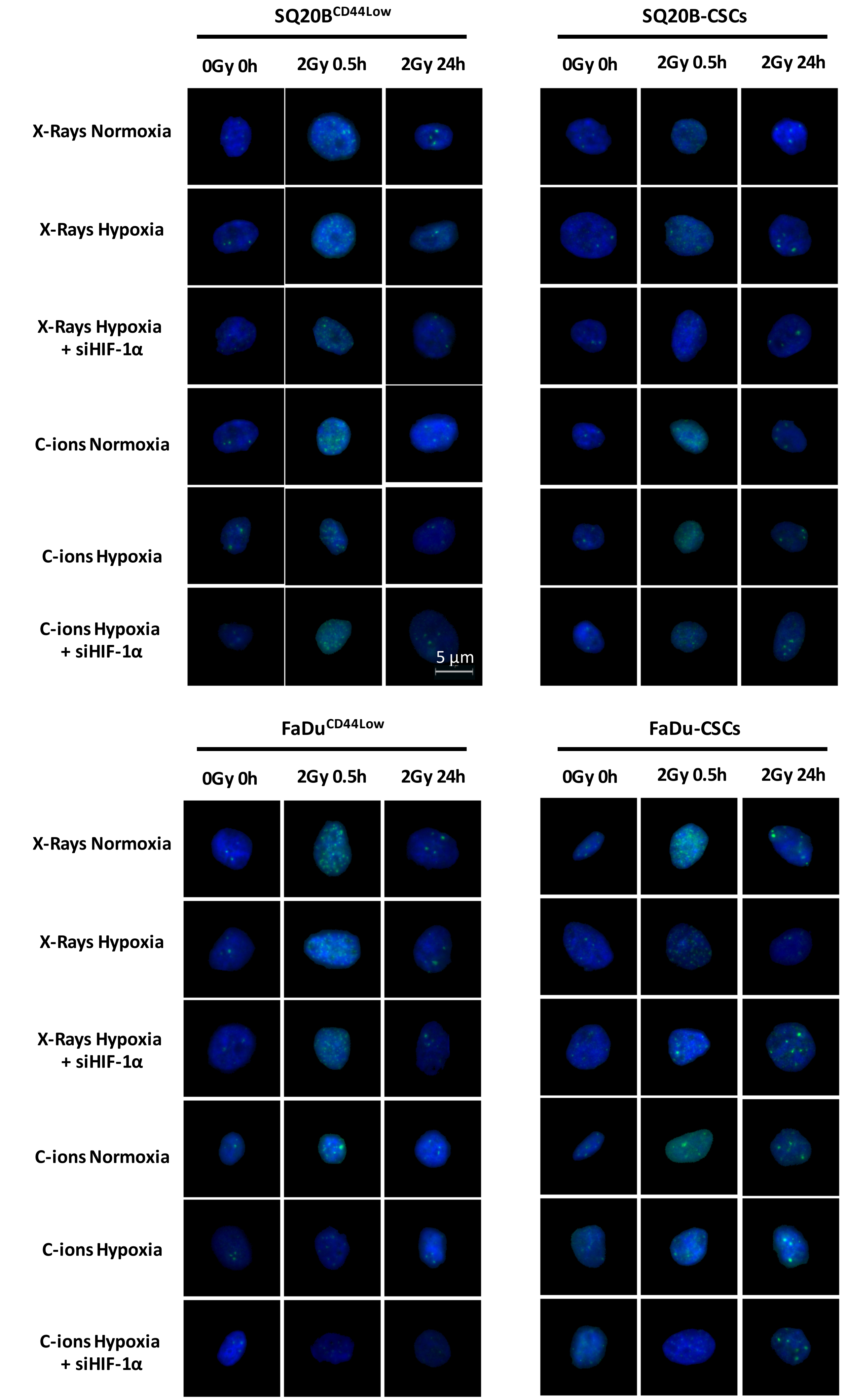

Supplementary Figure S7: *Representative images of P-DNA-PKcs foci per nucleus in SQ20B^{CD44Low}, FaDu^{CD44Low}, SQ20B-CSCs and FaDu-CSCs.*

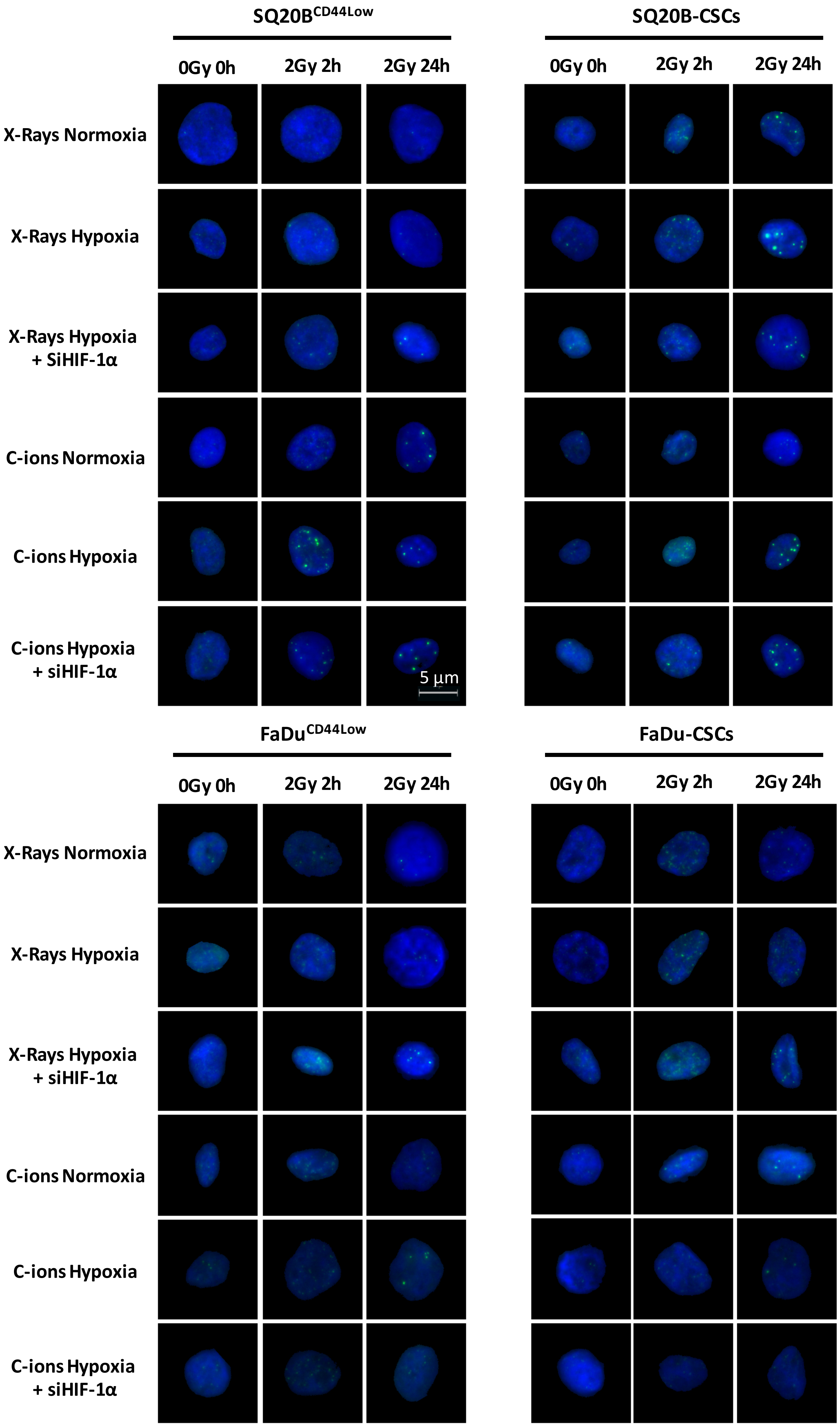

Supplementary Figure S8: *Representative images of RAD51 foci per nucleus in SQ20B^{CD44Low}, FaDu^{CD44Low}, SQ20B-CSCs and FaDu-CSCs.*
