# Supplementary material for: Involvement of HIF-1α in the Detection, Signaling, and Repair of DNA Double-Strand Breaks after Photon and Carbon-Ion Irradiation
Source: Cancers (Basel). 2021 Jul 30;13(15):3833. doi: 10.3390/cancers13153833 (PMC8345054; doi:10.3390/cancers13153833)
Supplement: Supplementary file 1 [file cancers-13-03833-s001.zip › Supplementary figures proof.pptx]

## Slide 1
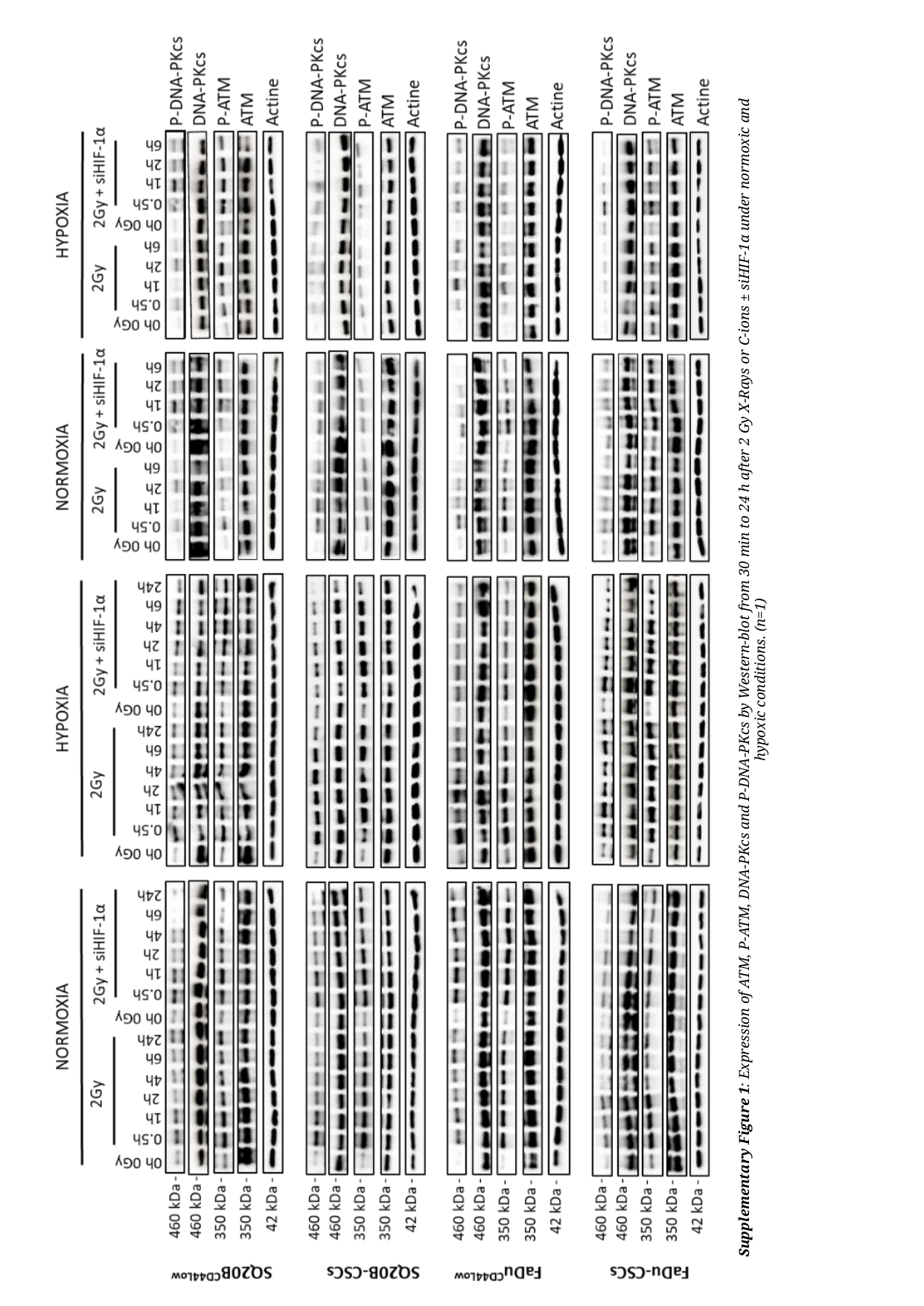

Supplementary Figure 1: Expression of ATM, P-ATM, DNA-PKcs and P-DNA-PKcs by Western-blot from 30 min to 24 h after 2 Gy X-Rays or C-ions ± siHIF-1α under normoxic and hypoxic conditions. (n=1)

## Slide 2
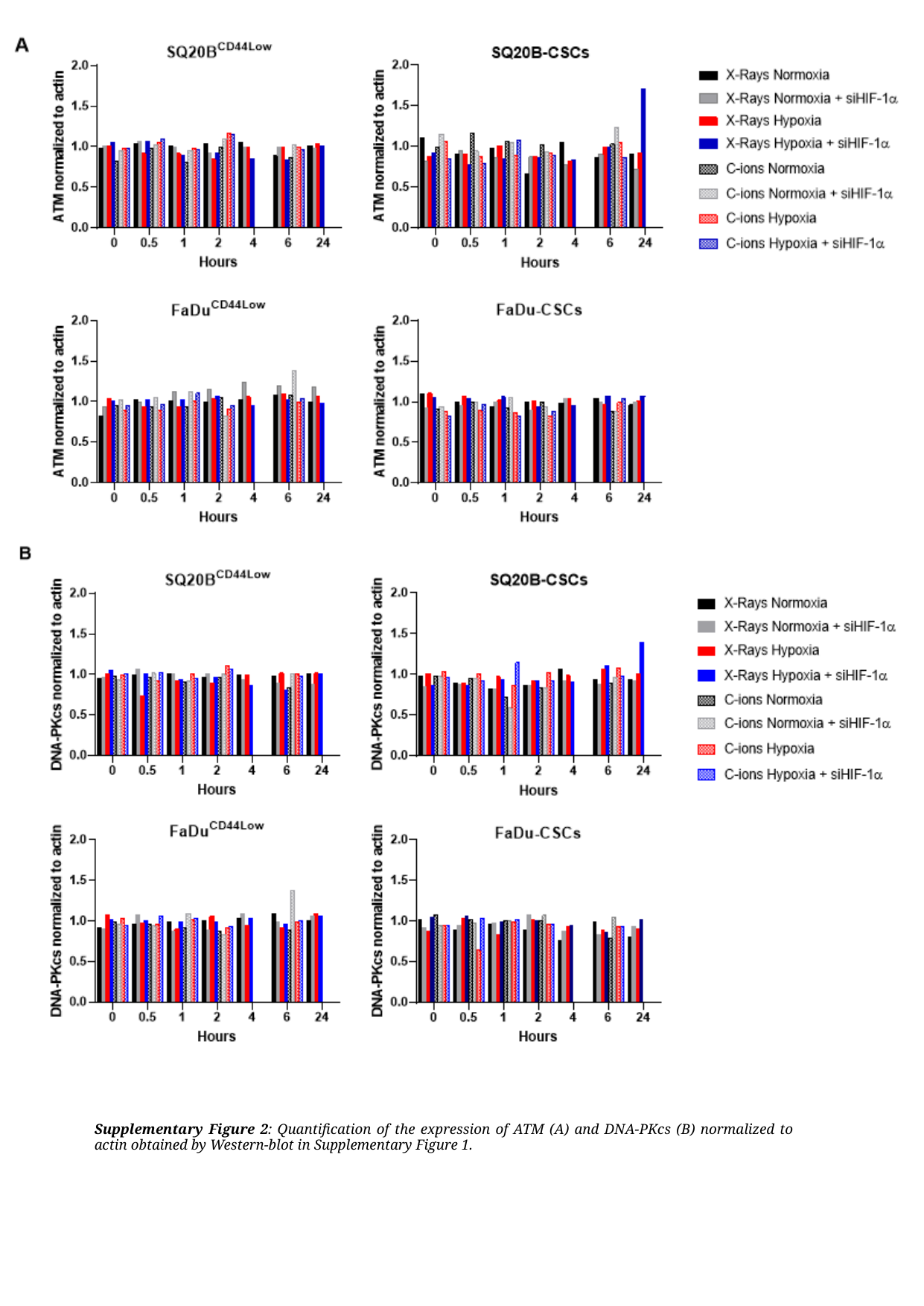

Supplementary Figure 2: Quantification of the expression of ATM (A) and DNA-PKcs (B) normalized to actin obtained by Western-blot in Supplementary Figure 1.

## Slide 3
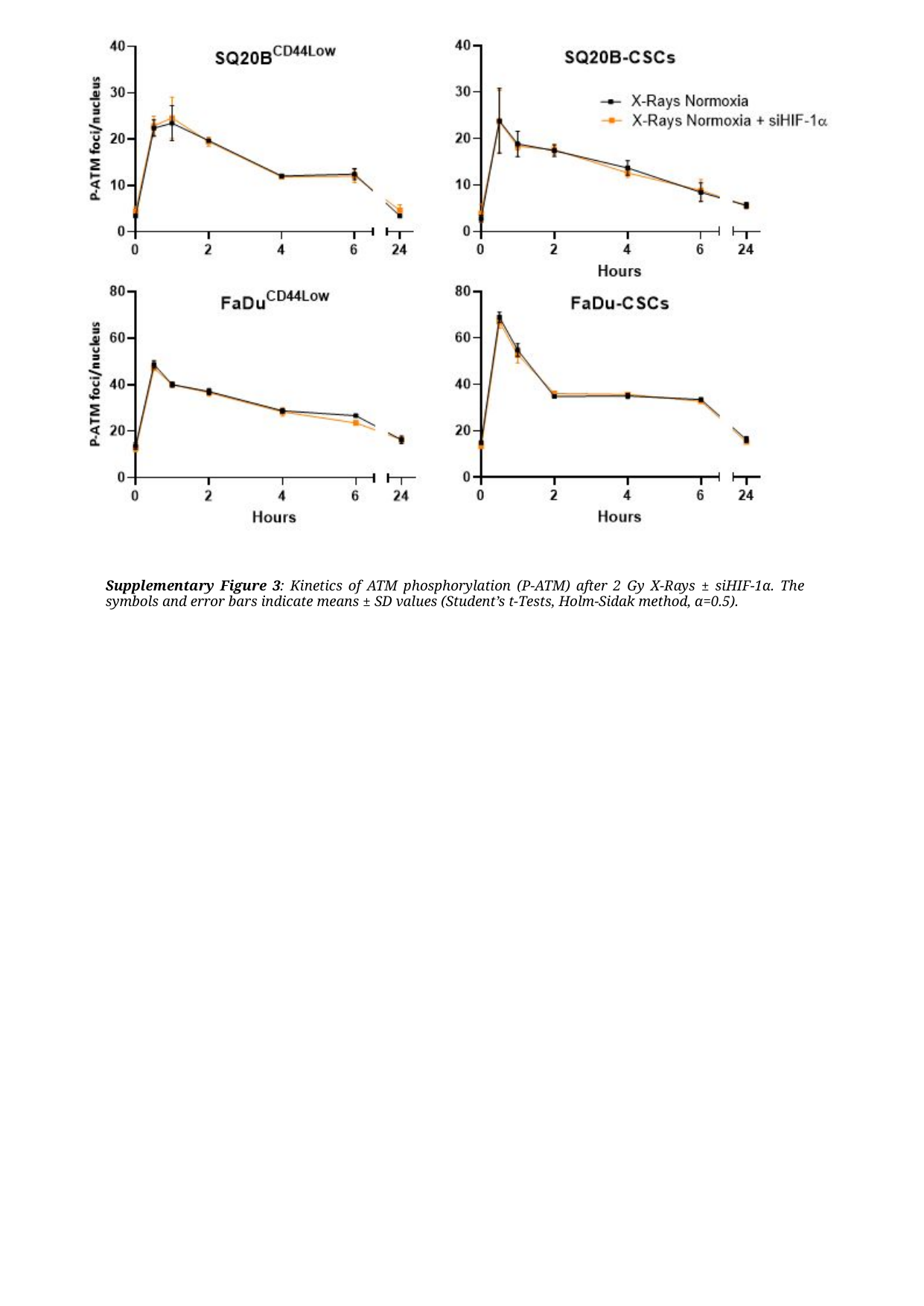

Supplementary Figure 3: Kinetics of ATM phosphorylation (P-ATM) after 2 Gy X-Rays ± siHIF-1α. The symbols and error bars indicate means ± SD values (Student’s t-Tests, Holm-Sidak method, α=0.5).

## Slide 4
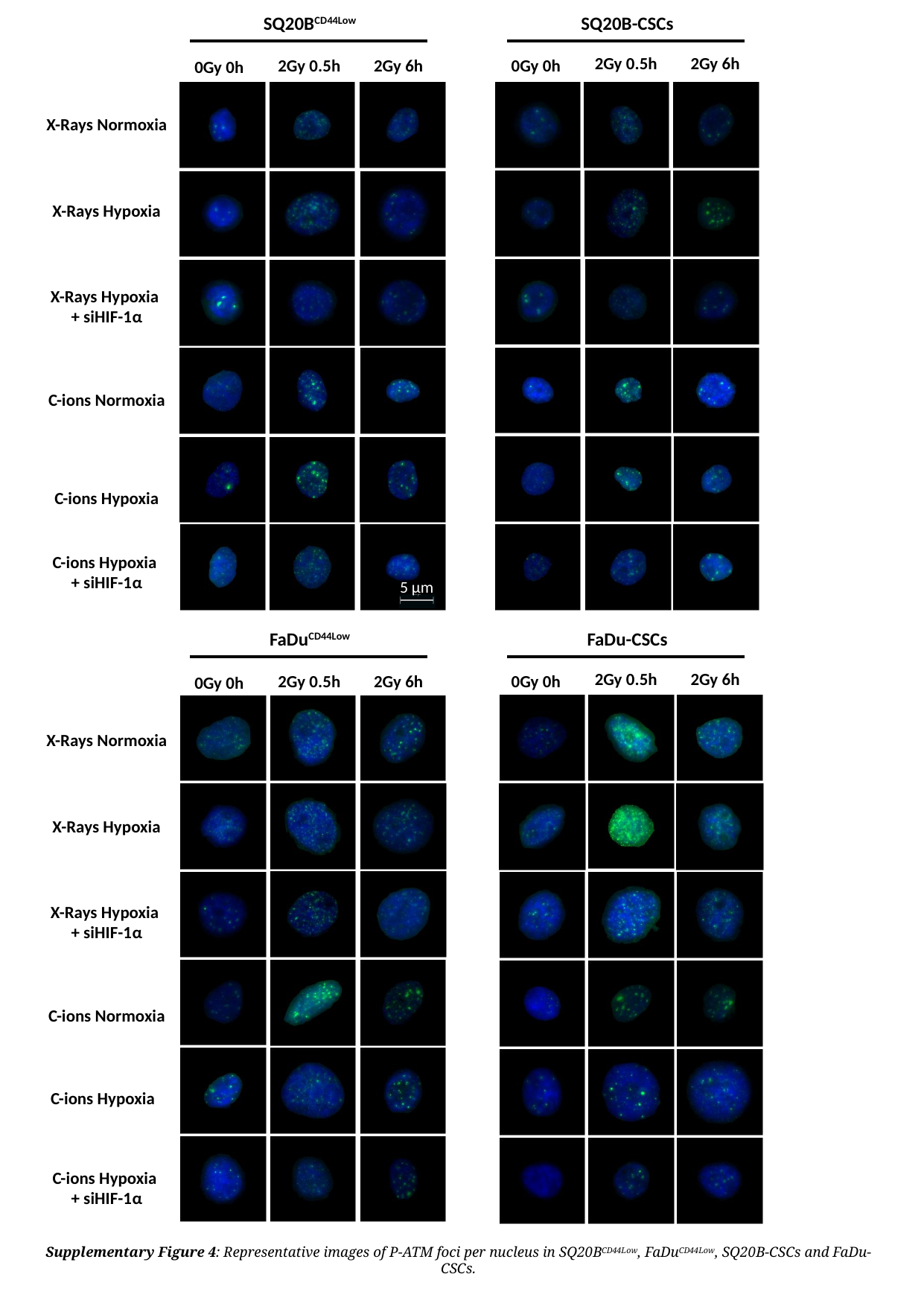

SQ20BCD44Low
SQ20B-CSCs
2Gy 0.5h
2Gy 6h
0Gy 0h
2Gy 0.5h
2Gy 6h
0Gy 0h
X-Rays Normoxia
X-Rays Hypoxia
X-Rays Hypoxia
+ siHIF-1α
C-ions Normoxia
C-ions Hypoxia
C-ions Hypoxia
+ siHIF-1α
5 µm
FaDuCD44Low
FaDu-CSCs
2Gy 0.5h
2Gy 6h
2Gy 0.5h
2Gy 6h
0Gy 0h
X-Rays Normoxia
X-Rays Hypoxia
X-Rays Hypoxia
+ siHIF-1α
C-ions Normoxia
C-ions Hypoxia
C-ions Hypoxia
+ siHIF-1α
0Gy 0h
Supplementary Figure 4: Representative images of P-ATM foci per nucleus in SQ20BCD44Low, FaDuCD44Low, SQ20B-CSCs and FaDu-CSCs.

## Slide 5
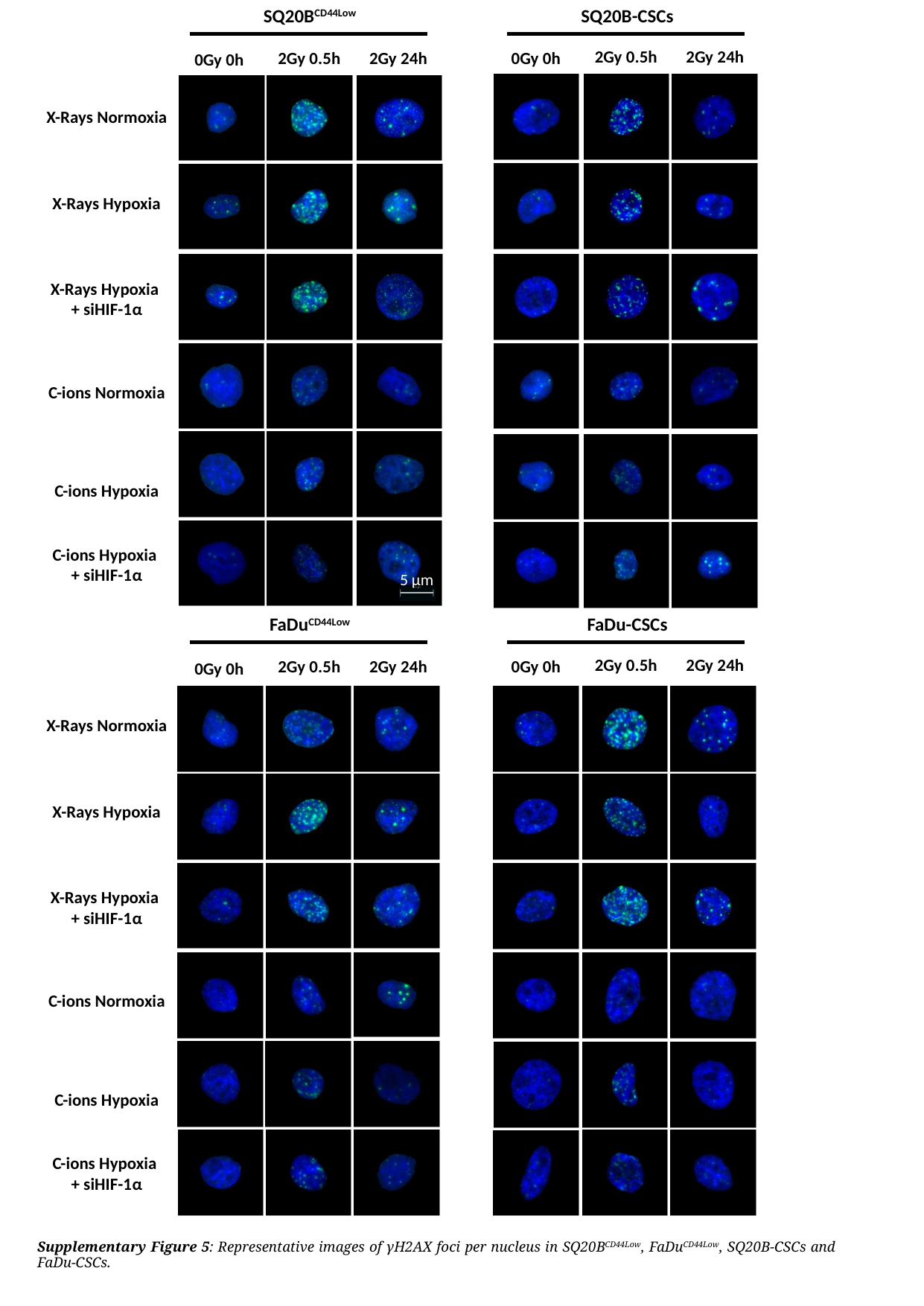

SQ20BCD44Low
SQ20B-CSCs
2Gy 0.5h
2Gy 24h
0Gy 0h
2Gy 0.5h
2Gy 24h
0Gy 0h
X-Rays Normoxia
X-Rays Hypoxia
X-Rays Hypoxia
+ siHIF-1α
C-ions Normoxia
C-ions Hypoxia
C-ions Hypoxia
+ siHIF-1α
5 µm
FaDuCD44Low
FaDu-CSCs
2Gy 0.5h
2Gy 24h
2Gy 0.5h
2Gy 24h
0Gy 0h
X-Rays Normoxia
X-Rays Hypoxia
X-Rays Hypoxia
+ siHIF-1α
C-ions Normoxia
C-ions Hypoxia
C-ions Hypoxia
+ siHIF-1α
0Gy 0h
Supplementary Figure 5: Representative images of γH2AX foci per nucleus in SQ20BCD44Low, FaDuCD44Low, SQ20B-CSCs and FaDu-CSCs.

## Slide 6
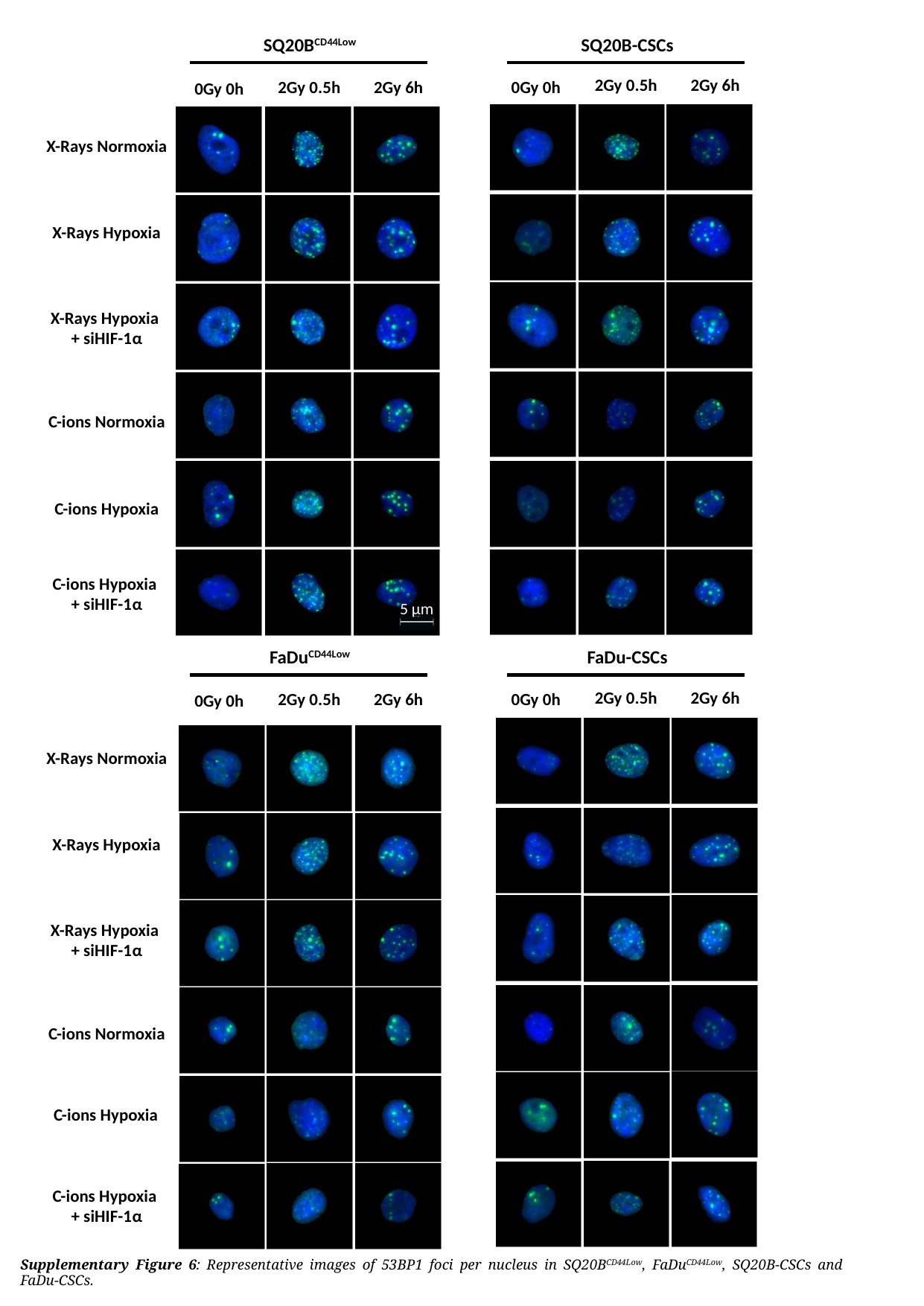

SQ20BCD44Low
SQ20B-CSCs
2Gy 0.5h
2Gy 6h
0Gy 0h
2Gy 0.5h
2Gy 6h
0Gy 0h
X-Rays Normoxia
X-Rays Hypoxia
X-Rays Hypoxia
+ siHIF-1α
C-ions Normoxia
C-ions Hypoxia
C-ions Hypoxia
+ siHIF-1α
5 µm
FaDuCD44Low
FaDu-CSCs
2Gy 0.5h
2Gy 6h
2Gy 0.5h
2Gy 6h
0Gy 0h
X-Rays Normoxia
X-Rays Hypoxia
X-Rays Hypoxia
+ siHIF-1α
C-ions Normoxia
C-ions Hypoxia
C-ions Hypoxia
+ siHIF-1α
0Gy 0h
Supplementary Figure 6: Representative images of 53BP1 foci per nucleus in SQ20BCD44Low, FaDuCD44Low, SQ20B-CSCs and FaDu-CSCs.

## Slide 7
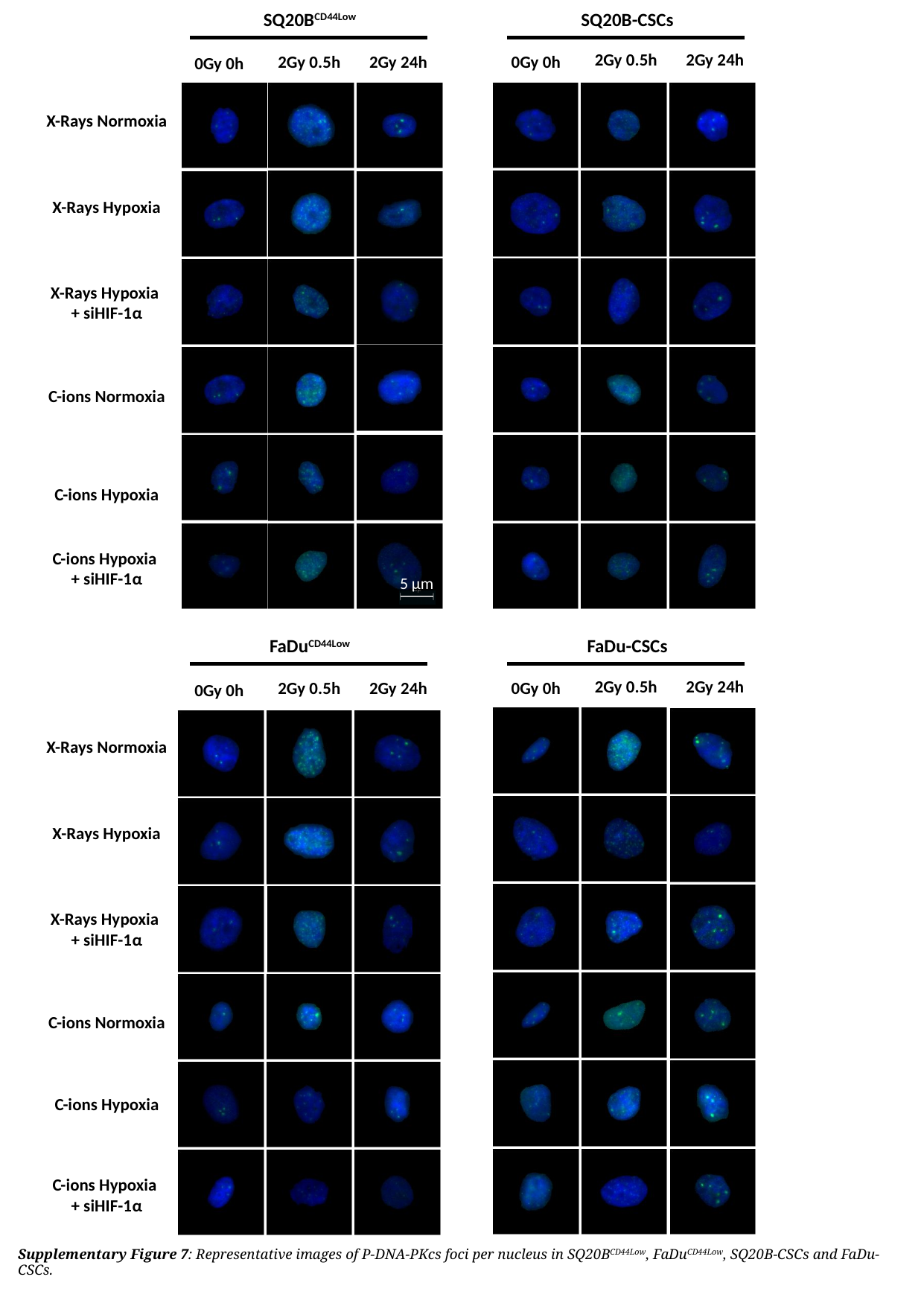

SQ20BCD44Low
SQ20B-CSCs
2Gy 0.5h
2Gy 24h
0Gy 0h
2Gy 0.5h
2Gy 24h
0Gy 0h
X-Rays Normoxia
X-Rays Hypoxia
X-Rays Hypoxia
+ siHIF-1α
C-ions Normoxia
C-ions Hypoxia
C-ions Hypoxia
+ siHIF-1α
5 µm
FaDuCD44Low
FaDu-CSCs
2Gy 0.5h
2Gy 24h
2Gy 0.5h
2Gy 24h
0Gy 0h
X-Rays Normoxia
X-Rays Hypoxia
X-Rays Hypoxia
+ siHIF-1α
C-ions Normoxia
C-ions Hypoxia
C-ions Hypoxia
+ siHIF-1α
0Gy 0h
Supplementary Figure 7: Representative images of P-DNA-PKcs foci per nucleus in SQ20BCD44Low, FaDuCD44Low, SQ20B-CSCs and FaDu-CSCs.

## Slide 8
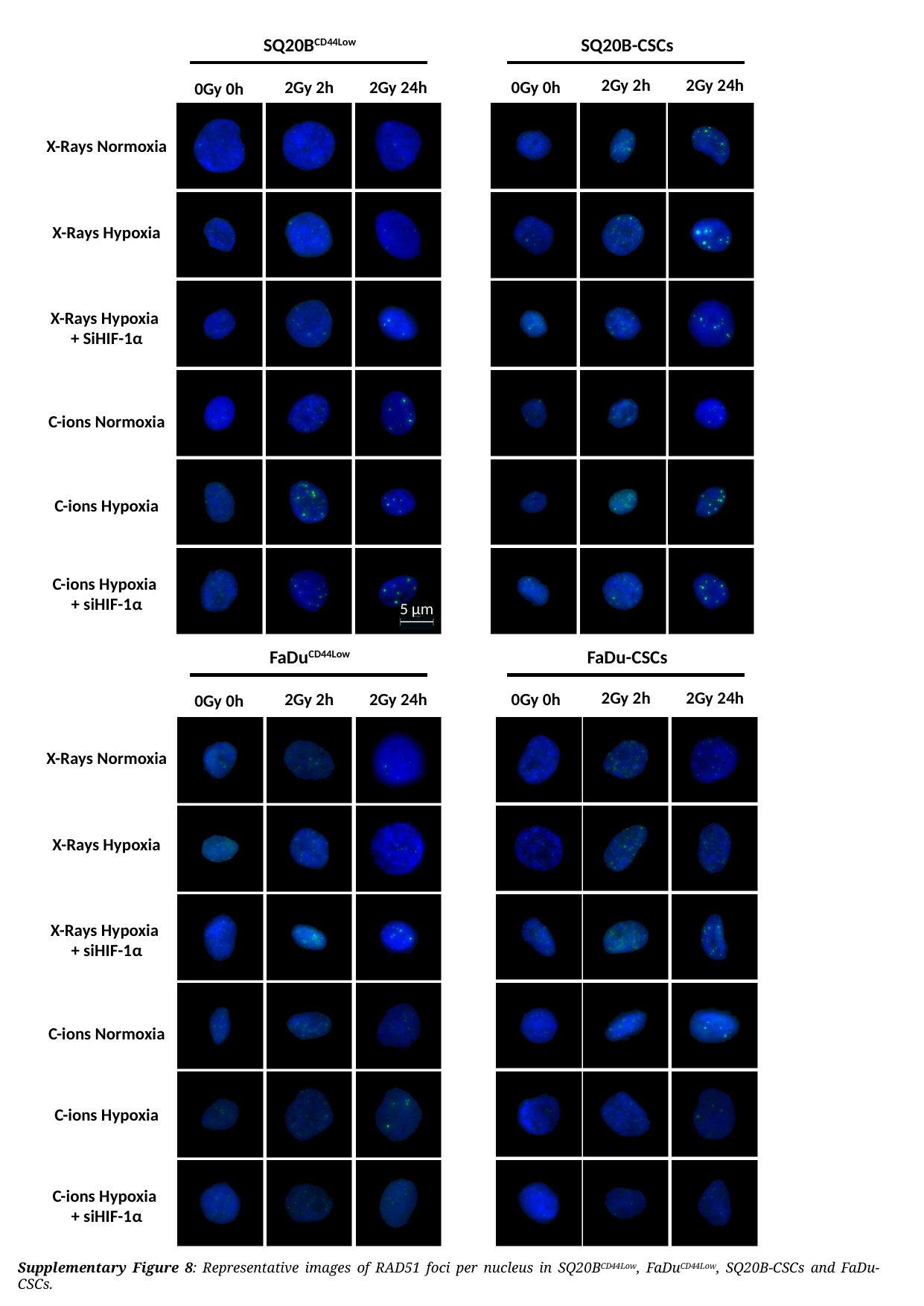

SQ20BCD44Low
SQ20B-CSCs
2Gy 2h
2Gy 24h
0Gy 0h
2Gy 2h
2Gy 24h
0Gy 0h
X-Rays Normoxia
X-Rays Hypoxia
X-Rays Hypoxia
+ SiHIF-1α
C-ions Normoxia
C-ions Hypoxia
C-ions Hypoxia
+ siHIF-1α
5 µm
FaDuCD44Low
FaDu-CSCs
2Gy 2h
2Gy 24h
2Gy 2h
2Gy 24h
0Gy 0h
X-Rays Normoxia
X-Rays Hypoxia
X-Rays Hypoxia
+ siHIF-1α
C-ions Normoxia
C-ions Hypoxia
C-ions Hypoxia
+ siHIF-1α
0Gy 0h
Supplementary Figure 8: Representative images of RAD51 foci per nucleus in SQ20BCD44Low, FaDuCD44Low, SQ20B-CSCs and FaDu-CSCs.
