# Supplementary material for: Involvement of HIF-1α in the Detection, Signaling, and Repair of DNA Double-Strand Breaks after Photon and Carbon-Ion Irradiation
Source: Cancers (Basel). 2021 Jul 30;13(15):3833. doi: 10.3390/cancers13153833 (PMC8345054; doi:10.3390/cancers13153833)
Supplement: Supplementary file 1 [file cancers-13-03833-s001.zip › Supplementary text Proof.pdf]

Supplementary text

HIF-1α does not increase ATM and DNA-PKcs levels but induces their phosphorylation

First, we investigated whether the effects of the silencing of HIF-1α on the DSB detection and the activation of the NHEJ-c pathway resulted from the phosphorylation of the ATM and DNA-PKcs proteins or an important recruitment of these proteins. The expressions of ATM and DNA-PKcs, as well as of their phosphorylated forms (P-ATM and P-DNA-PKcs), were followed by Western-blot for SQ20B^{CD44Low}, SQ20B-CSCs, FaDu^{CD44Low}, and FaDu-CSCs cells, in response to photon and C-ion irradiations, under normoxia and hypoxia, after the silencing or not of HIF-1α (Supplementary Figure S1). The expressions of the total amount of the ATM and DNA-PKcs proteins were quantified and normalized to actin for all the conditions tested (Supplementary Figure S2). Altogether, these quantifications underline that the recruitments of the ATM and DNA-PKcs proteins were not affected by the experimental conditions for the four populations. However, variations of P-ATM and P-DNA-PKcs were displayed starting 30 minutes after irradiation. Since ATM and DNA-PKcs proteins are constitutively expressed, respectively in the cytoplasm and the plasma membrane, the variations of the phosphorylation observed by Western-blot could not be specific from the DSB repair process into the nucleus. Consequently, the phosphorylation of ATM and DNA-PKcs studied by Western-blot are indicative but not specific from the DNA-repair process. To investigate the specific role of HIF-1α on the DNA-repair, we then determined the kinetics of induction and repair of the DSBs by fluorescent microscopy only into the cell nucleus, where DSBs are induced by irradiation.
